# Metabolic cross-feeding interactions modulate the dynamic community structure in microbial fuel cell under variable organic loading wastewaters

**DOI:** 10.1371/journal.pcbi.1012533

**Published:** 2024-10-17

**Authors:** Natchapon Srinak, Porntip Chiewchankaset, Saowalak Kalapanulak, Pornpan Panichnumsin, Treenut Saithong

**Affiliations:** 1 Bioinformatics and Systems Biology Program, School of Bioresources and Technology, School of Information Technology, King Mongkut’s University of Technology Thonburi (Bang Khun Thian), Bangkok, Thailand; 2 Center for Agricultural Systems Biology (CASB), Systems Biology and Bioinformatics research laboratory, Pilot Plant Development and Training Institute, King Mongkut’s University of Technology Thonburi (Bang Khun Thian), Bangkok, Thailand; 3 Excellent Center of Waste Utilization and Management, National Center for Genetic Engineering and Biotechnology, National Sciences and Technology Development Agency at King Mongkut’s University of Technology Thonburi, Bangkok, Thailand; US Army Medical Research and Materiel Command: US Army Medical Research and Development Command, UNITED STATES OF AMERICA

## Abstract

The efficiency of microbial fuel cells (MFCs) in industrial wastewater treatment is profoundly influenced by the microbial community, which can be disrupted by variable industrial operations. Although microbial guilds linked to MFC performance under specific conditions have been identified, comprehensive knowledge of the convergent community structure and pathways of adaptation is lacking. Here, we developed a microbe-microbe interaction genome-scale metabolic model (mmGEM) based on metabolic cross-feeding to study the adaptation of microbial communities in MFCs treating sulfide-containing wastewater from a canned-pineapple factory. The metabolic model encompassed three major microbial guilds: sulfate-reducing bacteria (SRB), methanogens (MET), and sulfide-oxidizing bacteria (SOB). Our findings revealed a shift from an SOB-dominant to MET-dominant community as organic loading rates (OLRs) increased, along with a decline in MFC performance. The mmGEM accurately predicted microbial relative abundance at low OLRs (L-OLRs) and adaptation to high OLRs (H-OLRs). The simulations revealed constraints on SOB growth under H-OLRs due to reduced sulfate-sulfide (S) cycling and acetate cross-feeding with SRB. More cross-fed metabolites from SRB were diverted to MET, facilitating their competitive dominance. Assessing cross-feeding dynamics under varying OLRs enabled the execution of practical scenario-based simulations to explore the potential impact of elevated acidity levels on SOB growth and MFC performance. This work highlights the role of metabolic cross-feeding in shaping microbial community structure in response to high OLRs. The insights gained will inform the development of effective strategies for implementing MFC technology in real-world industrial environments.

## Author summary

Microbial fuel cells (MFCs) use microorganisms to break down waste biomass and produce electricity directly. The technology holds potential for sustainably efficient wastewater treatment that supports the concept of green economy and zero-waste society. However, application of MFCs to the actual industrial wastewaters was shown a decline in electricity generation and treatment efficiency when organic loading rates (OLR) increased. This decline of MFC performance was attributed to alterations in microbial composition. The lack of understanding regarding the impact of OLR on microbial composition has hindered the maintenance of optimal performance and has consequently impeded the practical application of MFC systems. To this end, we developed a metabolic model (GEM) of microbial community (called mmGEM) to investigate the metabolic cross-feeding interactions which is an important mechanism affecting microbial composition dynamics. Simulation of mmGEM showed that the change of common metabolite cross-feedings altered the microbial composition during increasing OLR. Further analysis of mmGEM offer practical recommendations for optimizing MFCs by supporting growth and interaction of electricity-related microbial guild. In addition, our work exhibits the use of mmGEM to simulate scenario-based circumstances of microbial communities in MFCs that effectively facilitate an optimization of MFC to real-world implementation in industrial sector.

## Introduction

Microbial fuel cells (MFCs) are an ideal technological solution to support zero-waste practices within the concept of a circular economy [1]. This technology exploits microbial activities to extract electrons from the breakdown of waste biomass. These electrons can be released to anode electrode and then flow to cathode electrode for completing the circuit and generating electricity [2]. MFCs offer distinct advantages over existing green technologies such as anaerobic digestion, including the ability to directly convert organic wastes into electricity, the capability to remove toxic residues, and the production of less-polluting effluents [3]. Thus, MFCs can complement the anaerobic digestion process or serve as an alternative green technology for waste treatment across diverse industries [4]. A recent study on the application of MFCs in wastewater treatment systems within the canned-pineapple industry underlines the practicality of this technology in real-world scenarios. The MFCs system showed great performances in chemical oxygen demand (COD) removal, sulfide removal, and in current generation with organic- and sulfide-containing wastewater [5]. Nonetheless, the performance of MFCs is often hindered by microbial activity destabilization when exposed to fluctuating conditions of real-world industrial operations [6]. The variable nature of organic substrates in wastewater profoundly influences the microbial dynamics within MFCs, including the composition, metabolic functions, and interrelationships among microbial guilds, thereby ultimately impacting their performance. Different waste characteristics favor the growth of specific microbial species, causing a shift in microbial profiles and composition [7]. Moreover, microorganisms do not live in isolation but interact through substrate-product exchanges. This exchange of metabolites known as “*metabolic cross-feeding*” drives intricate interactions between microbes, which finally modulate the convolution of the microbial community [8–10]. While there is extensive research on how microbial species influence each other and the sensitivity of microbial communities to environmental conditions, our current understanding of the fundamental mechanisms underlying these interactions and variations remains limited.

Microbial species in MFCs typically cooperate through mutualistic interactions (i.e., syntrophic relationship), where they rely on each other for survival and perform complementary roles in converting organic matter into electricity. Complex carbon compounds are primarily digested into smaller carbon molecules, such as volatile fatty acids (VFAs), ethanol, acetate, and hydrogen (H_2_), by organic-hydrolyzing and organic-fermenting microbes. These molecules are subsequently oxidized by exoelectrogenic bacteria (EB), which are capable of performing extracellular electron transfer to produce electricity [11]. In sulfur-rich environments, sulfate-reducing bacteria (SRB) play a major role in metabolizing sulfate and larger carbon molecules. This metabolism results in the production of smaller carbon e.g. VFAs, which support the growth of various bacteria, including sulfide-oxidizing bacteria (SOB), which utilize VFAs and, in turn, provide sulfate as a substrate for SRB [12,13]. Additionally, prevalence of specific substrates such as acetate, formate, and H_2_ in MFCs tend to favor methanogenic bacteria (MET) [14,15]. Growth of MET helps scavenge the remaining substrates, thus maintaining COD removal efficiency [16]. The interactions among microbial guilds are highly dynamic and largely affected by the surrounding environment, including substrate availability and concentration [17,18]. These conditions pose an even higher challenge in pursuing a comprehensive study and manipulation of MFCs performance.

Wastewater characteristics, including substrate type, concentration, and pH vastly influence microbial interactions and composition within MFCs. Different types of organic substrates can selectively support the growth and activity of distinct microbial species. For example, acetate-fed MFCs exhibited a high abundance of *Geobacter* species, while glucose-fed MFCs favored the prevalence of *Clostridium* and *Bacillus* bacteria [10,19]. Besides substrate species, the composition of each individual organic compounds in the industrial wastewater varies with the production seasons, and these fluctuations alter the organic loading rate (OLR) to MFCs. Studies have shown that an increase in OLR in MFCs can lead to a decline in electricity generation. This deterioration is likely due to the invasion of MET, which compete against EB in MFCs [15,16]. Changes in substrate types and concentration, coupled with the MFC system operation, impact the pH levels in the reactor chamber. This pH alteration critically disrupts the equilibrium of the microbial community and the performance of the system. An acidic environment can decrease the overall growth rate of the microbial community, impacting electricity generation. In addition, it can alter the overall activity and structure of the microbial community as different species may respond variably to pH changes, leading to alterations in their behavior and organization within the community [20,21]. In real-world situations, the equilibrium of microbial interactions faces challenges from multiple influential factors. These combined factors increase the complexity of the interrelationships between species.

The intricate and diverse relationships among microbial species and their surrounding environments prevent in-depth investigation of metabolic cross-feeding in MFCs. While metabolic flux analysis using ^13^C labeling can precisely track microbial metabolism [22], it has limitations in capturing the multitude of metabolite exchanges within microbial populations. Moreover, metabolic flux analysis is primarily applicable in synthetic laboratory settings [23–25]. To address these challenges, mathematical modeling has been introduced to study microbe-microbe interactions in MFCs [25–28]. Artificial intelligence (AI)-based modeling is a powerful approach to accommodate a large number of microbial species that are involved in the scope of study [26], whereas metabolic modeling is a niche approach to gain comprehensive insights underlying the association of microbial species in a community [25,28]. In particular, the genome-scale metabolic models (GEMs) of microbial communities created using Flux Balance Analysis (FBA) framework [29] allow the observation of mechanistic scenarios of microbial interactions under any studied conditions that are not restricted to the realm of empirical experiments [30–33]. The GEMs approach is typically conceived as an integrated compartmental model to represent the fundamental metabolic process generated by the entire microbial community, or as a compartmentalized model to mimic the complementation and restriction inter-relationships between individual metabolic processes provided by the community members [34]. Previous studies of the compartmentalized GEMs demonstrate the ideality of the conceptual framework for examining how the microbial structure as a whole may adapt to changing conditions due to modified interactions between microorganisms. The multi-species GEM was initially adopted to deepen understanding of the commensal relationship between SRB and MET in a co-culture experiment, where hydrogen exchange was found to be a key metabolic cross-feeding that supports their syntrophic growth [35,36]. The approach has been improved in a number of ways to enable the simulation of the complex connections between microbial species in the real-world situation, for examples incorporation of microbial ecology constraints [37], contextualization of community dynamics through integration of omics data [38], and inclusion of spatial and temporal effects on microbial interactions and composition under dynamic environments [39–41]. Despite significant progress, the compartmentalized GEMs model still faces challenges in maximizing the number of community members in model coverage and minimizing computational load resulting from the large number of complexly associated components in order to arrive at a mathematically plausible solution. Addressing these challenges would improve the model’s representation of community-level scenarios.

In this work, we aimed to address a critical gap in the practical implementation of industrial MFCs by focusing on understanding the dynamics of microbial communities under changing operating conditions, particularly an increase in the OLR. We postulated that microbial interactions, specifically *metabolic cross-feeding* through substrate-product exchange, would be disrupted during elevated OLR conditions. This disruption might favor different microbial guilds, resulting in alternative equilibria of cross-feeding, where different microbial species interact with each other in new ways to exchange nutrients and metabolites. Consequently, these shifts could lead to changes in the overall composition of the microbial community, with some species becoming more dominant while others decline. To this end, we developed an effective modeling approach of microbe-microbe interaction GEM, or mmGEM, based on a functional-based lumped compartmentalized structural model design of the main microbial guilds that hold essential metabolic capabilities required for MCF substrate conversion under context of study. Our approach fundamentally intends to minimize the computational complexity of modeling while preserving the highest level of complexity present in real-world scenarios. This design compromises ideas between the compartmentalized and lumped metabolic model by representing microbial compartment of a metabolism-specific function by assuming that each essential metabolic function is not driven by only a single species but by a cohort sharing functional characteristics. This approach allows an inclusion of microbial species while limiting the number of compartments in the model. Expanding the community model also improves its representativeness by accounting for a larger proportion of the nutrients available in the real condition. In particular, the mmGEM was employed to represent the metabolic cross-feeding interactions among SRB, MET, and SOB microbial guilds in MFCs under varying OLRs. The model demonstrated a transition of a SOB-dominant community in low production seasons (L-OLR) to a MET-dominant community in high production seasons (H-OLR) from previous experiments, with the simulated cross-feeding dynamics. Despite of simplified microbial guilds, the model displayed mechanism of metabolic cross-feeding transition influencing the change of microbial community composition. Interestingly, enhancing SRB-SOB interactions could save SOB-dominant communities from collapsing under increasing OLR conditions. Leveraging our understanding, we proposed scenario-based mmGEM simulations incorporating elevated environmental H^+^ concentrations to improve the growth of SOB, which expected to improve MFC performance under high OLR condition. Indeed, we demonstrated the potential of leveraging GEMs in MFC applications. These models facilitate the study of MFCs and the implementation of eco-friendly technology in real industrial settings.

## Results

### Metabolic cross-feeding model of microbial community in MFC

**Fig 1:**
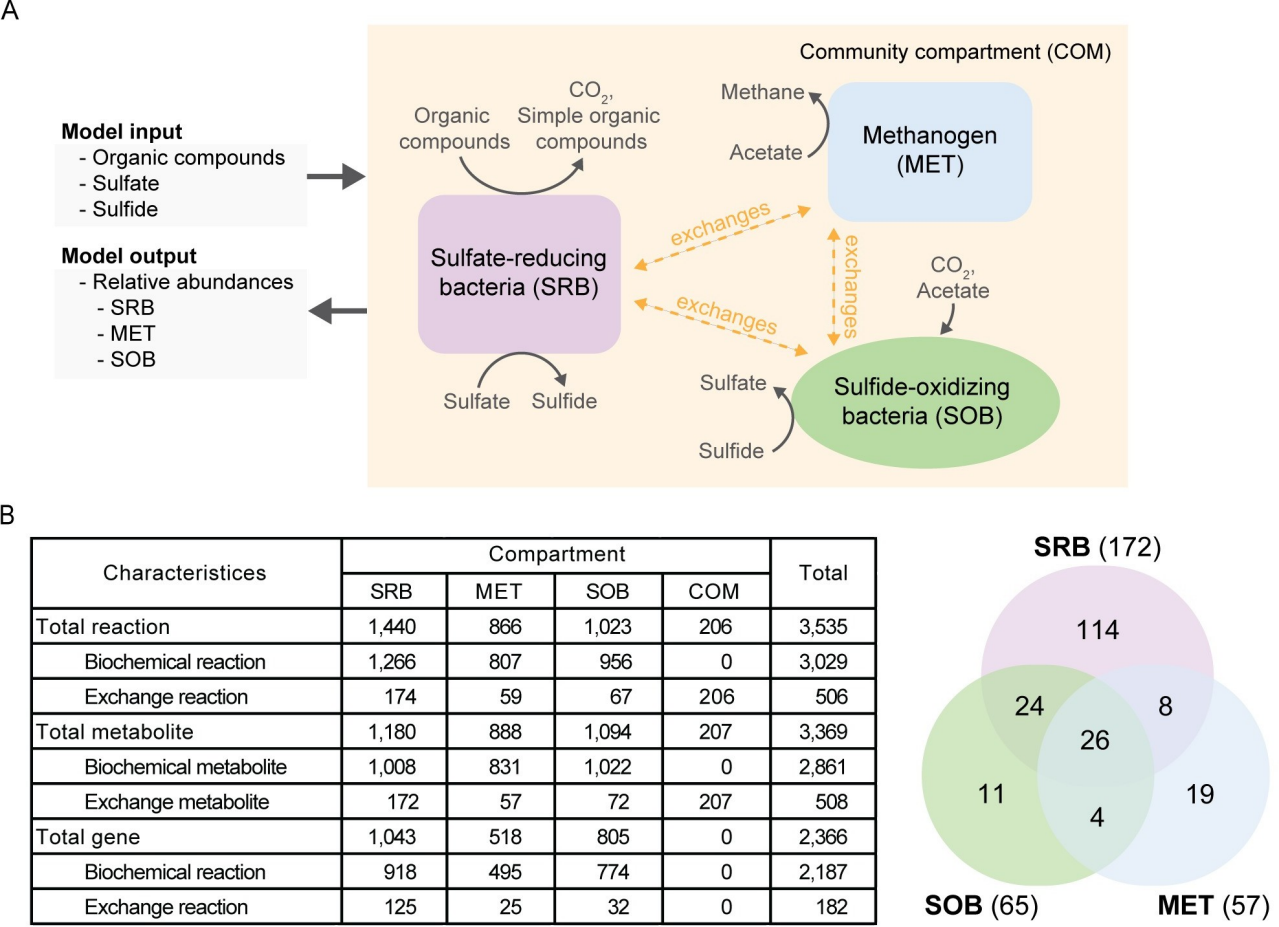
Design and characteristics of mmGEM. **A**, Conceptual design of microbe-microbe interaction model (mmGEM) for simulating microbial association in MFC community. The mmGEM is a compartmentalized model consisting of microbial guild compartments (including sulfate-reducing bacteria (SRB), methanogens (MET), and sulfide-oxidizing bacteria (SOB)), and the community compartment (COM). The scheme also depicts the main substrate uptake (model inputs) and product generation (model outputs) of the studied community. **B**, Characteristics of the mmGEM model encompass the numbers of biochemical reactions, metabolites, and relevant metabolic genes. Venn diagram shows the number of exchange metabolites for each individual microbial guild and the number of common exchange metabolites that infers their cross-feeding association.

The performance of the MFC system treating sulfide-containing wastewater from a canned-pineapple factory declined as the organic loading increased during high-production seasons (S1A Fig). Sriwichai et al. (2024) likewise demonstrated that the performance of MFCs is impacted by changes in the microbial community structure, including variations in the composition and relative abundance profiles, at high organic loading conditions. A genome-scale model of microbe-microbe interactions (mmGEM; S1 File) was developed to comprehensively study the associations among microbial species in the MFC ecosystem, particularly focusing on how the community adapts to variable OLR conditions. Given the high complexity and diversity of microbial community in MFCs, the mmGEM was designed using an effective modelling approach based upon the functionally-lumped compartmentalized structure, which extends microbial and environmental coverages while maintaining computational demand and model reconstruction effort. This approach assumes that each fundamental metabolic function essential for substrate conversion in MFC is not driven by a single species, but rather by a cohort sharing metabolic characteristics. Thus, a microbial compartment in the mmGEM conceptually incorporated multiple microbial species, serving as functional guild representative. This allowed the mmGEM to simulate the microbial interaction underlying the phenomenon based on the aggregated microbial compartment of a metabolism-specific function while accounting larger proportion of microbial species and environmental condition (see also S1 Text). The model was also built on the assumptions that: 1) the equilibrium state of the ecosystem is mainly modulated by the predominant microbial cohorts and 2) these groups grow inter-dependently based on substrate-product cross-feeding and their ability to compete for available substrates within a common pool. Fig 1A illustrates the hypothetical metabolic cross-feeding model involving SRB, MET, and SOB, which are three major microbial groups identified in MFC ecosystems (S1 Table [5]). The associations among these microbial groups are driven by their individual metabolic characteristics [13,42,43], and the wastewater composition profile (S1 Fig). Each group represents essential metabolic functions required to achieve the overall metabolic activity observed in MFC performance. We hypothesized that as MFC environments transition to high organic loading, the composition of organic substrates changes, thereby disrupting the equilibrium of substrate uptake and product secretion among major microbial groups (S1 Text). This disturbance influences the community structure by altering growth-dependent substrate availability and creating uneven growth potentials in competitive substrate conditions. The model first simulated the microbial interactions influencing the relative abundance of the microbial community in MFCs operated under low OLR (L-OLR) conditions during periods of low seasonal production (S1 Fig). Subsequently, the model assessed how microbial metabolic cross-feeding responded to high OLR (H-OLR) conditions (S1 Fig).

**Fig 2:**
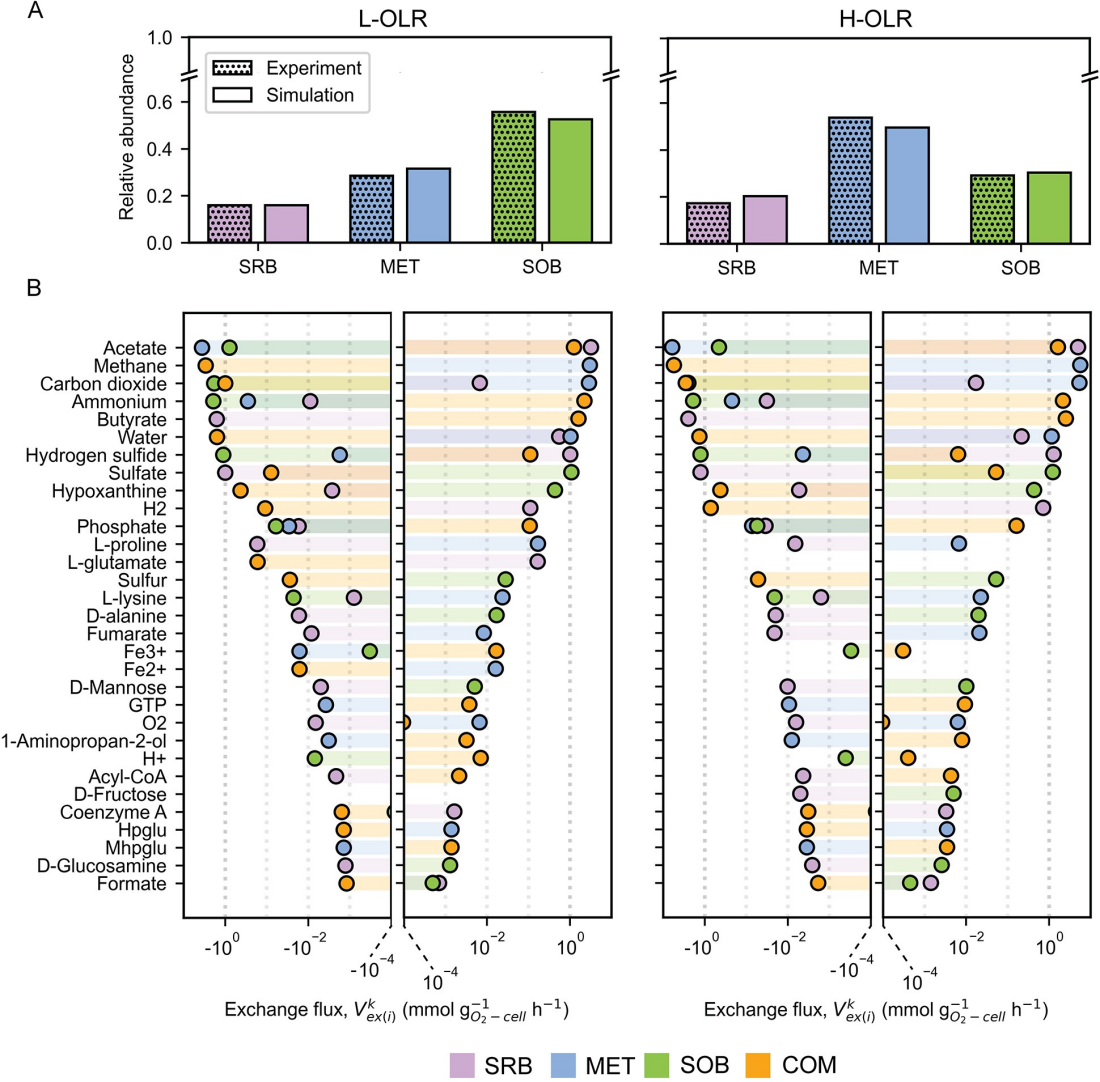
Simulation of metabolic cross-feeding in L-OLR and H-OLR conditions. **A**, Comparison of microbial relative abundances from experiments and microbe-microbe interaction model (mmGEM) simulations. **B**, The main metabolite exchange fluxes ($V_{ex(i)}^{k}$) between the key microbial guilds, including sulfate-reducing bacteria (SRB), methanogens (MET) and sulfide-oxidizing bacteria (SOB), and community compartment (COM) under low (L-OLR) and high organic loading conditions (H-OLR). $V_{ex(i)}^{k}$ is an exchange reaction flux of the metabolite *i* in COM and the microbial group *k*, including SRB, MET, and SOB. Positive (+) and negative (-) exchange fluxes indicate metabolite secretion (+) and consumption (-) for microbial compartments, while representing metabolite influx (+) and efflux (-) for COM. Exchange fluxes on the x-axis are represented on an exponential scale.

The mmGEM comprised four spatial compartments representing three microbial groups (i.e. SRB, MET, and SOB), and one common pool of metabolites (community compartment—COM) (Fig 1A). The model finally contained 3,535 reactions related to 3,369 metabolites, 2,366 metabolic genes (Fig 1B (Table) and S2 Table). These microbial groups freely exchanged organic substrates and metabolites with each other within the COM space (See also S2 Table and S2 Text). The SRB group had the highest number of exchangeable metabolites, followed by SOB and MET. The Venn diagram in Fig 1B presents a set of common metabolites potentially exchanged between the microbial groups, indicating cross-feeding. There were 62 metabolites shared between at least two microbial compartments, 26 of which, including acetate, sulfide, H^+^, CO_2_, and NH_4_, were common in all three microbial compartments. SRB and SOB shared the most exchangeable metabolites (50 common metabolites), indicating a strong cross-feeding association compared to the interactions among SRB-MET (34 common metabolites) and SOB-MET (30 common metabolites). Subsequently, the mmGEM was parameterized based on the exchange capacity ($v_{ex(i)}^{k}$) of major metabolites related to energy metabolism including acetate, sulfide, and H^+^, to match the measured relative abundances of microbes in L-OLR conditions (S2 Text). Finally, the mmGEM successfully simulated the microbial profile under L-OLR conditions (S1 Fig) accurately replicating the relative abundances of SRB, MET and SOB with error percentages of 0.31, 10.79, and 5.61, respectively, and 5.57 on average (Fig 2A, left). Moreover, the model estimated the maximum acetate consumption capacity of SOB (lower boundary (LB) of $v_{ex(Acetate)}^{SOB}$) to be approximately -1.50 mmol $g_{O_{2}‐\mathrm{cell}}^{‐1}\;h^{‐1}$, while the minimum acetate consumption capacity of MET (upper boundary (UB) of $v_{ex(Acetate)}^{MET}$) was about -11.55 mmol $g_{O_{2}‐\mathrm{cell}}^{‐1}\;h^{‐1}$. These results suggest that MET metabolized acetate at least 7 folds higher than SOB in this environment. In addition, the model indicated a maximum sulfide-producing capacity from SRB (UB of $v_{ex(Sulfide)}^{SRB}$) of around 6.28 mmol $g_{O_{2}‐\mathrm{cell}}^{‐1}\;h^{‐1}$, and recommended maintaining low level of H^+^ exchange capacity of SRB and MET (UB of $v_{{ex(H}^{+})}^{SRB}$ and $v_{{ex(H}^{+})}^{MET}$) at approximately $1\times 10^{‐5}\;\mathrm{mmol}\;g_{O_{2}‐\mathrm{cell}}^{‐1}\;h^{‐1}$ to prevent excessive H^+^-scavenging by SOB (S2 Fig). In this context, the function-based representation in mmGEM not only simulated the microbial structure of the major cohort but also provided deeper insights into microbial interactions under specific conditions, which are often difficult to capture due to limited measured data.

### Simulation of microbial interactions in low and high organic loading conditions

The amount of organic material entering the MFC system varied significantly between production seasons, with a 14-fold increase in OLRs from “low season” (L-OLR, 103 ${\mathrm{mg}}_{O_{2}}\;h^{‐1}$) to “high season” (H-OLR, 1,430 ${\mathrm{mg}}_{O_{2}}\;h^{‐1}$). The huge increase in OLRs not only resulted in higher concentrations of organic substrates but also altered the ratio of organic to inorganic components in the MFC influents. Specifically, there were notable changes in the levels of acetate, butyrate, sulfate, and sulfide. S1 Fig shows that butyrate loading increased the most by approximately 1.56 times, followed by a 1.30-times increase in acetate. This unequal increment shifted the C4-to-C2 substrate composition ($V_{ex(Butyrate)}^{COM}/V_{ex(Acetate)}^{COM}$) ratio from 1.29 to 1.54, indicating a higher dominance of butyrate in H-OLR conditions. In addition to carbon substrates, there was an inverse shift in the sulfate-to-sulfide between both OLR conditions. This suggests a shift in the overall metabolic state from sulfate accumulation to reduction in the MFC system, possibly resulting from either a change in the net sulfide oxidation to net sulfate reduction or a decrease in sulfate levels due to intense sulfide oxidation to sulfur. This change in metabolic activity is postulated to be a result of the adjusted metabolic cross-feeding in response to the higher OLR environment.

The mmGEM simulated substrate utilization and cross-feeding of SRB, SOB and MET, reflecting their relative abundance in L-OLR conditions (Fig 2A, left). The results showed that these microbial guilds exchanged metabolites, mainly butyrate, acetate, sulfate, sulfide, NH_4_, methane, and CO_2,_ (Fig 2B, left), which served as key carbon and nitrogen sources and metabolic currency compounds involved in core biosynthesis processes of microbial metabolism, emphasizing the significance of syntrophic cross-feeding relationships within the MFC microbiome. The simulation of metabolite cross-feeding showed that SOB greatly benefitted from autotrophic CO_2_ fixation for their growth, consequently releasing high amounts of sulfate into the environment. The increasing sulfate levels, together with butyrate loading, provided substrates for SRB whose metabolism returned acetate, sulfide, and CO_2_ to the community. Despite being the only microbial group capable of directly utilize butyrate, a major organic component in wastewater influent, SRB exhibited minimal relative abundance compared to SOB and MET (Fig 2, left). The scenario underscores the strong dependency of SRB growth on sulfate, which functions as a final electron acceptor in anaerobic sulfate respiration. The presence of sulfate in the system, exclusively produced by SOB, was postulated to constrain the overall metabolic activity of SRB by limiting electron transfer. This constraint reduces the available energy necessary to fuel the complete biosynthesis process in SRB. This postulation is supported by the high secretion rates of acetate as an excess substrate ($V_{ex(Acetate)}^{SRB}/V_{ex(Acetate)}^{COM}$ = 2.5 times) and sulfide as a metabolic product ($V_{ex(Sulfide)}^{SRB}/V_{ex(Sulfide)}^{COM}$ = 9 times) by SRB, possibly for trading with sulfate, a growth-limiting substrate. In contrast to SRB and SOB, the Venn diagram in Fig 1B shows that MET has fewer common metabolic exchanges, suggesting that it may be more dependent on acetate, another major organic compound available in industrial wastewater from canned-pineapple. Simulation shows that MET consumed acetate over 4.5 times more than SOB ($V_{ex(Acetate)}^{MET}/V_{ex(Acetate)}^{SOB}$). It led to the relatively higher abundance of MET compared to SRB, but still lower than SOB who gain additional benefit from the CO_2_ produced by acetoclastic methanogenesis of MET’s metabolism. The simulations, in addition, demonstrated the cross-feeding of carbohydrates and amino acids among different microbial guilds; for instance, the transfer of L-proline, L-lysine, and fumarate from MET to SRB, and D-alanine, D-mannose, D-glucosamine, and hypoxanthine from SOB to SRB. Since MET and SOB rely on SRB for their growth, this mutual relationship likely exists to maintain the fitness and functionality of the primary producer [44].

Furthermore, the mmGEM-simulated cross-feeding interactions among SRB, SOB and MET were assessed the model’s ability to predict the microbial profile under elevated OLR conditions. As shown in Fig 2A (right), the mmGEM was able to mimic the response of the microbial structure to H-OLRs (S1 Fig). The relative abundance of SRB, SOB and MET was correctly estimated, with an average error percentage of 9.89. This demonstrated that the mmGEM based on functional compartmentalized structure could simulate the shift in equilibrium from an SOB-dominant to MET-dominant state during the transition from L-OLR to H-OLR, as shown in Fig 2A. Additionally, the model simulations provide interactions for each microbial guild that explain the change of community structure. The rise in OLR mostly impacted microbial metabolite exchanges by augmenting flux quantities, as indicated by the levels of butyrate and acetate in the COM (Fig 2B, right). In H-OLR conditions, MET was the most abundant microbial guild, surpassing SOB, while SRB remained the least abundant. The increase in acetate availability in H-OLR conditions favored the metabolism of MET guild, as evidenced by their 13.5 times higher acetate consumption rate than SOB ($V_{ex(Acetate)}^{MET}/V_{ex(Acetate)}^{SOB}$). The high competitiveness of MET may originate from their relatively more efficient acetoclastic process and overall dependence on acetate as a carbon source. In contrast, SOB growth was strongly interdependent on SRB, whose metabolism was tightly tied to sulfide-sulfate exchange, masking their growth under elevated OLRs. In this condition, the relative abundance of SOB reduced in correlation with acetate consumption. This resulted in only slightly higher sulfide-sulfate exchange with SRB despite an excessive amount of butyrate. Additionally, acetate, H_2_, and formate secreted by SRB appeared to be relatively higher under high OLR conditions. These results imply the inter-limiting growth of these microbial guilds under this environment, and the appearing constraint on the SOB guild does not seem to be compensated by the autotrophic CO_2_ fixation process.

### Metabolic adaptation of individual microbial guilds contributes to overall cross-feeding in MFCs under variable OLRs

**Fig 3:**
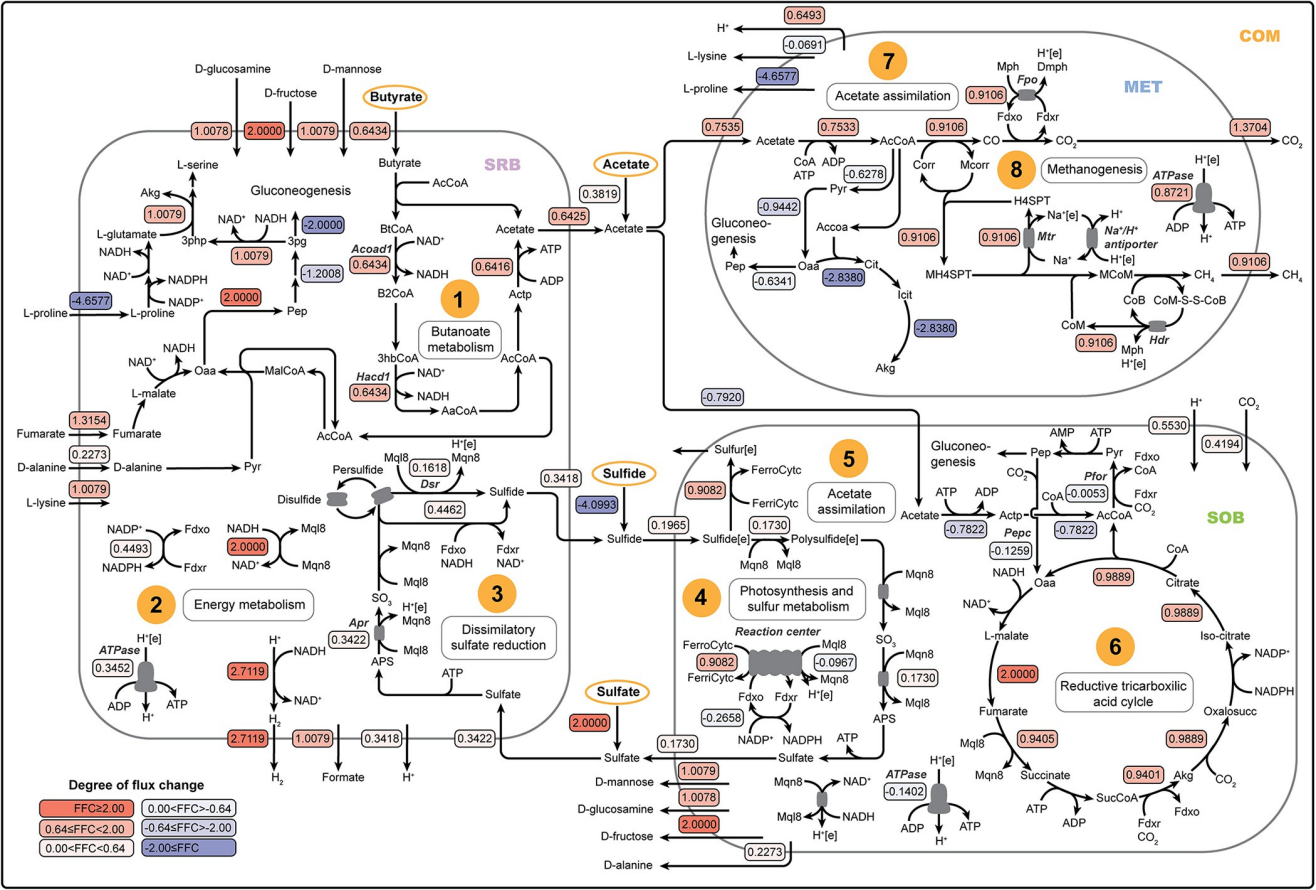
Differences in metabolic fluxes within the microbial community under H-OLR and L-OLR conditions. The major metabolic cross-feedings ($V_{ex(i)}^{k}$) and intracellular flux ($V_{j}^{k}$) differences are represented as flux fold changes (FFCs; denoted as a number in rounded rectangles). The color of red and blue represent positive (+) and negative (-) FFCs, indicating increase and decrease of a reaction flux change in H-OLR, respectively. Intensity levels represent the degree of change, categorized as low (0.00<|FFC|<0.64), medium (0.64≤|FFC|<2), and high (|FFC|≥2) relative to the uptake rate of butyrate (0.64 FFC). Metabolites marked with yellow circles represent model input (data-specific conditions). $V_{ex(i)}^{k}$ is exchange reaction flux of the metabolite *i* in community compartment (COM) and the microbial group *k* including sulfate-reducing bacteria (SRB), methanogen (MET), and sulfide-oxidizing bacteria (SOB). $V_{j}^{k}$ is intracellular reaction fluxes of reaction *j* in the microbial group *k* including SRB, MET, and SOB. Abbreviations: SRB = sulfate-reducing bacteria, MET = methanogens, SOB = sulfide-oxidizing bacteria, COM = community compartment, Acoad1 = acyl-CoA dehydrogenase, Hacd1 = 3-hydroxyacyl-CoA dehydrogenase, ATPase = ATP synthase, Apr = adenylylsulfate reductase, Dsr = dissimilatory sulfite reductase, Pfor = pyruvate:ferredoxin oxidoreductase, Pepc = phosphoenolpyruvate carboxylase, Fpo = F_420_H_2_ dehydrogenase, Mtr = methyl transferase, and Hdr = heterodisulfide reductase.

Alteration of microbial structure resulting from increased OLRs has been shown to be associated with metabolic cross-feeding among microbial guilds. Specifically, at H-OLRs, there was a notable increase in acetate utilization by MET, contrasting with the limited use of acetate by SOB. Also, SRB, whose relative abundance remained steady across conditions, exhibited a more productive metabolism by releasing proportionally larger amounts of acetate, sulfide, H_2_, and formate compared to the L-OLR conditions. Here, we further investigated the responses of metabolic conversion within microbial guilds and their influence on constraining the overall metabolic cross-feeding among associated microbes in MFC. Metabolic fluxes, which describe the specific rates of metabolic reactions in metabolism, were comparatively studied between H-OLR and L-OLR conditions, denoted as the log flux fold change (FFC) ($FFC={log}_{2}\left(\frac{V_{\left(H-OLR\right)}^{k}}{V_{\left(L-OLR\right)}^{k}}\right)$; see details in Materials and Methods). Fig 3 presents the core metabolic pathways with remarkably high FFCs within each microbial guild, along with the associated cross-feeding reactions. Positive FFC values (marked as red) indicate increasing changes in metabolic fluxes in H-OLR conditions, while negative FFCs (marked as blue) denote decreasing changes.

The simulated flux distribution within SRB highlighted constraints on microbial growth due to a shortage of sulfate, which is vital for sustaining respiration. This resulted not only in a deficiency of ATP for metabolic processes but also in alterations to the synthesis of metabolic byproducts. The uptake rate of butyrate was notably high (FFC = 0.6434) in wastewater with higher OLRs, and it was solely utilized by SRB through the butanoate metabolic pathway (FFC = 0.6434). This pathway converted butyrate to acetate, generating significant energy in the forms of ATP and NADH (Fig 3, Number 1). The ATP derived from the extended utilization of butyrate and cross-fed metabolites (amino acids and carbohydrates) led to an increase in the growth rate of SRB in H-OLR conditions. However, the observed growth of the SRB guild appeared to be lower than expected when considering the uptake of substrates. This discrepancy might be due to the inefficiency of SRB in utilizing NADH for the sulfate respiration pathway—the dissimilatory sulfate reduction pathway. First, NADH was converted to menaquinol (Mql8), which serves as a pivotal electron carrier essential for cellular respiration processes (Fig 3, Number 2). Subsequently, menaquinol actively participated in the sulfate respiration pathway, in which the electron transport chain facilitated the movement of electrons across a series of protein complexes, culminating in the reduction of sulfate to sulfide (Fig 3, Number 3). The limited utilization of NADH was compounded by the consequent constraint on sulfate uptake (FFC = 0.3422). The results showed that only 34% of NADH produced from butanoate metabolism was directed to the electron transport chain ($\left(V_{Apr}^{SRB}+V_{Dsr}^{SRB})/(V_{Acoad1}^{SRB}+V_{Hacd1}^{SRB}\right)$) in H-OLR conditions compared to around 44% in L-OLR conditions. The lack of sulfate in the electron transport chain resulted in deviations in the proton motive force (H^+^[e]) and the activity of ATPase reaction (FFC = 0.3452) (Fig 3, Number 2). The ineffective respiration directly affected the overall metabolic biosynthesis in SRB and suppressed cellular biomass production. SRB prioritized the use of high-energy molecules (ATP and NADH) for a variety of metabolic processes beyond just respiration and biomass biosynthesis. For instance, SRB allocated these molecules to support other metabolic pathways, such as the generation of formate (FCC = 1.0079) and H_2_ (FCC = 2.7119) (Fig 3, Number 2).

In a lack of sulfide from SRB, the energy metabolism and carbon assimilation in SOB were challenged due to substrate limitation, resulting in growth suppression. Given that mmGEM was permitted to freely exchange photons with the surroundings (S2 Text), the energy metabolism of SOB was impacted by reduced cross-feeding from SRB and the low concentration of sulfide in wastewater under H-OLR conditions. The limited availability of sulfide constrained sulfur metabolism (0.1730 FFC), thus preventing reduction reactions involving oxidizing agents such as Mqn8 during the conversion of sulfide to sulfate (Fig 3, Number 4). This resulted in a relatively lower proton gradient (H^+^[e]), reduced ferredoxin (Fdxr) levels, and a decrease in NADH derived from photosynthesis, culminating in a 10% decrease in the flux of ATPase (FFC = -0.1402) in SOB under H-OLR conditions (Fig 3, Number 4). The lower ATP production subsequently restricted carbon assimilation for biomass synthesis and microbial growth, mainly via acetate assimilation (FFC = -0.7920) and CO_2_ fixation. The CO_2_ fixation reactions showed negative FFCs for both phosphoenolpyruvate carboxylase (Pepc) which converts phosphoenolpyruvate (Pep) to oxaloacetate (Oaa) (FCC = -0.1259) and pyruvate:ferredoxin oxidoreductase (Pfor) which converts acetyl CoA (AcCoA) to pyruvate (Pyr) (FCC = -0.0053) (Fig 3, Number 5). The results also indicated the predominant role of acetate in SOB growth as shown by the negative FFC of Pfor (Fig 3, Number 6), and the increased activity of CO_2_ fixation through the reductive tricarboxylic acid (RTCA) pathway (FFC = ~0.90) could not compensate for the carbon assimilation from acetate metabolism. Moreover, it was observed that SOB tended to convert sulfide to sulfur (FFC = 0.9082) instead of fully oxidizing it to sulfate (FFC = 0.1730). This preference was further indicated by an increase in the ratio of sulfur secreted to sulfide uptake fluxes, which rose from 2.5% in L-OLR conditions to 4.2% in H-OLR conditions. Consequently, this resulted in low sulfate accumulation in the MFC, corresponding to the decrease in sulfate content in H-OLR conditions (S1 Fig).

The decline in interactions between SRB and SOB, along with the low dependency on acetoclastic methanogenesis, facilitated the proliferation of MET and their dominance over other microbial guilds under H-OLR conditions. MET capitalized on the ample supply of acetate, resulting from both elevated wastewater concentrations and reduced acetate assimilation by SOB, under H-OLR conditions. This scenario favored MET due to acetate’s dual role as both a carbon and energy source. The heightened activity of acetate assimilation (FFC = 0.7535) supported the acetoclastic methanogenesis pathway by enabling the establishment of a proton gradient (H^+^[e]) across cellular membranes. Key enzymes such as heterodisulfide reductase (Hdr), F_420_H_2_ dehydrogenase (Fpo), and methyl transferase (Mtr) facilitated this process. Consequently, the proton gradient led to increased production of ATP (FFC = 0.8721) (Fig 3, Number 7 and 8). In addition, this pathway contributed to higher secretions of CO_2_ (FFC = 1.3704) and methane (FFC = 0.9106), collectively indicating MET’s effectiveness in adapting to H-OLR conditions. These results suggest that MET efficiently utilized acetate for growth, outcompeting other microbial groups, especially SOB. Moreover, MET’s robust acetoclastic methanogenesis was crucial in eliminating COD, which explains the maintenance of COD removal efficiency above 70% despite a significant drop in current density (~35% reduction) under H-OLR conditions (S1 Fig).

### Prediction of metabolic cross-feeding dynamics and MFC microbial profiles under varying butyrate and acetate loadings

The mmGEM simulation suggested that the concentration of organic substrates, especially butyrate and acetate, played a prominent role in driving metabolic cross-feedings and shaping the microbial community composition. Metabolic cross-feeding among SRB, MET, and SOB, the major microbial guilds in MFCs, effectively described their growth interrelationships in response to varying OLRs. This was particularly evident through the utilization of butyrate, acetate, sulfate, sulfide, CO_2_, H_2_ and formate. Based on the relationships among the MFC microbial guilds, mmGEM was used to forecast tentative alterations in microbial cross-feedings under varying butyrate and acetate loadings, as well as the resulting microbial profiles in the MFC system. In this analysis, the mmGEM model was used to simulate the metabolic exchange capacity ($v_{ex(i)}^{k}$), to infer the specific conversion abilities of microbial reactions within their metabolism. This was observed through a linear increase in community-level butyrate uptake (from 0.48 to 3.61 mmol $g_{O_{2}‐\mathrm{cell}}^{‐1}\;h^{‐1}$) and acetate uptake (from 0.77 to 2.09 mmol $g_{O_{2}‐\mathrm{cell}}^{‐1}\;h^{‐1}$), extending across L-OLR and H-OLR conditions (S3 Table). The analysis subsequently highlighted the capacity of functional compartmentalized metabolic model, mmGEM, to simulate dynamic behaviors of environment and intricate interaction capacity of microbial guild.

**Fig 4:**
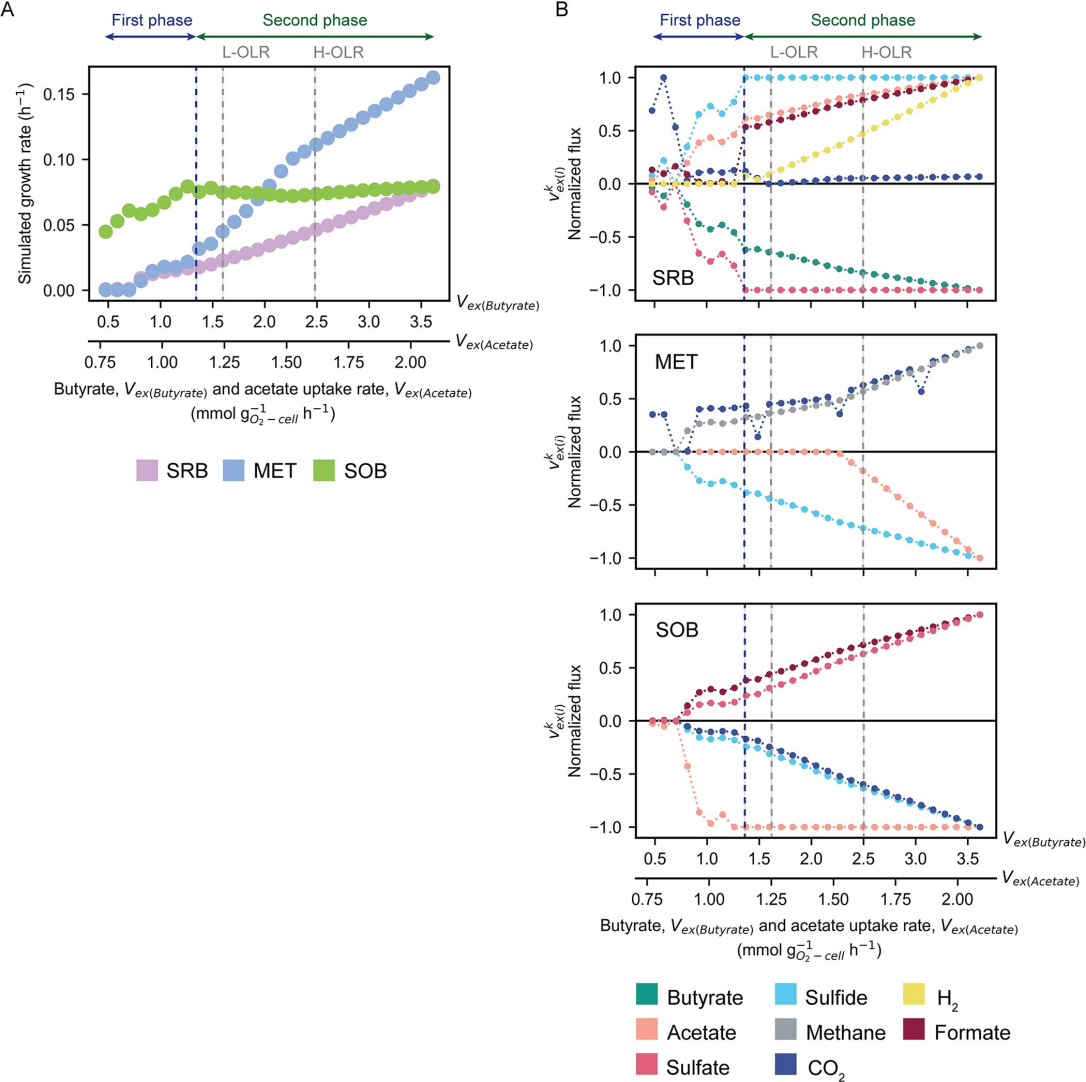
Simulated microbial growth rate and metabolic cross-feeding adaption under increasing organic concentration. **A**, Microbial growth rates and **B**, exchange capacity fluxes ($v_{ex(i)}^{k}$) of sulfate-reducing bacteria (SRB), methanogens (MET), and sulfide-oxidizing bacteria (SOB) under increasing organic concentration. The fluxes of an exchange capacity across the increasing organic concentration range were normalized using min-max normalization. The first and second phases are separated by a dashed-blue line. The first phase is defined between 0.48 and 1.26 mmol $g_{O_{2}‐\mathrm{cell}}^{‐1}\;h^{‐1}$ for butyrate uptake rate and 0.77 to 1.10 mmol $g_{O_{2}‐\mathrm{cell}}^{‐1}\;h^{‐1}$ for acetate uptake rate. The second phase ranges from 1.37 to 3.61 and 1.15 to 2.09 mmol $g_{O_{2}‐\mathrm{cell}}^{‐1}\;h^{‐1}$ for butyrate and acetate uptake rates, respectively. Dashed-grey lines represent points of the organic concentration, equal to low organic loading condition (L-OLR) and high organic loading conditions (H-OLR). $v_{ex(i)}^{k}$ is exchange capacity flux of the metabolite *i* of microbial group *k* including SRB, MET, and SOB. Positive (+) and negative (-) normalized exchange capacity fluxes indicate metabolite secretion (+) and consumption (-) for microbial compartments.

The results showed that the growth of microbial guilds within the MFC system varied depending on the quantity of butyrate and acetate loaded into the system (Fig 4A). At low levels of butyrate (0.48–1.26 mmol $g_{O_{2}‐\mathrm{cell}}^{‐1}\;h^{‐1}$) and acetate (0.77 to 1.10 mmol $g_{O_{2}‐\mathrm{cell}}^{‐1}\;h^{‐1}$) uptakes, SOB dominated, while the growth of MET and SRB was comparable (Fig 4A, First phase). When the organic content was higher, about 1.37–3.61 mmol $g_{O_{2}‐\mathrm{cell}}^{‐1}\;h^{‐1}$ for butyrate and 1.15–2.09 mmol $g_{O_{2}‐\mathrm{cell}}^{‐1}\;h^{‐1}$ for acetate, SRB and particularly MET kept growing at a higher rate in response to the increased availability of substrates. In contrast, the growth rate of SOB appeared to remain constant, and SOB seemed to lose their ability to effectively compete for and utilize the increased substrates (Fig 4A, Second phase). The inability of SOB to effectively utilize substrates contributed to the dominance of MET in environments with higher concentrations of organic compounds. Specifically, MET thrived in high acetate environments due to their efficient metabolism and growth capability. In addition, SRB growth increased during the second phase and converged to the same maximal rate as the growth of SOB, which is related to their tight relationship in sulfate-sulfide exchange.

We further analyzed the metabolite exchange capacity fluxes under dynamic OLRs to observe the key metabolic cross-feedings driving the transition towards an alternative community equilibrium in H-OLR conditions. The analysis revealed that major metabolite exchanges—such as acetate exchange and sulfide-sulfate (S) cycling between SRB and SOB, caused growth limitations in SOB and changed the overall community composition. The simulation indicated that limited S cycle between SRB and SOB corresponded with the constant growth of SOB. The butyrate uptake capacity in SRB increased with the organic content, while sulfide secretion and sulfate uptake capacities reached their maximum (normalized flux = 1) at the beginning of the second phase (Fig 4B, SRB). These maximal flux capacities constrained sulfide secretion from keeping pace with increased organic uptakes, as indicated by a reduced slope during the second phase (S3 Fig, SRB). The disproportional sulfide secretion inhibited the growth of SOB by directly limiting their energy source (Figs 4A and S3 (SOB)), while prompting SRB to produce H_2_ and formate (S3 Fig, SRB). Furthermore, the acetate exchange capacity of SOB correlated with the sulfide secretion capacity of SRB. This correlation sustained the growth of SOB, with their acetate exchange capacity peaking towards the end of the first phase (Fig 4B, SOB). We observed that even though the acetate uptake of SOB declined, the increasing CO_2_ and sulfide uptake fluxes helped maintain their growth (S3 Fig, SOB). These CO_2_ and sulfide uptakes compensated for acetate, as suggested by their elevated flux capacities in the second phase (Fig 4B, SOB). However, this autotrophic growth was not enough to enhance the growth rate of SOB and compete with MET during increasing organic uptakes. The results collectively suggest that the degenerated SRB-SOB interaction hampered the growth of SOB during the second phase, allowing MET to thrive owing to their highly efficient acetoclastic methanogenesis. The minimal acetate uptake capacity during the initial organic perturbation consolidated their efficient metabolism (Fig 4B, MET), while the rapid increase in the growth rate of MET (Fig 4A) gradually shaped the overall community structure. In addition, the simulated results suggest that MET could support SRB and exhibit co-growth during the second phase (Fig 4A). In fact, the continuous butyrate metabolism in SRB was supported by the persistent acetate utilization of MET (S3 Fig, MET), maintaining a steady-state condition.

**Fig 5:**
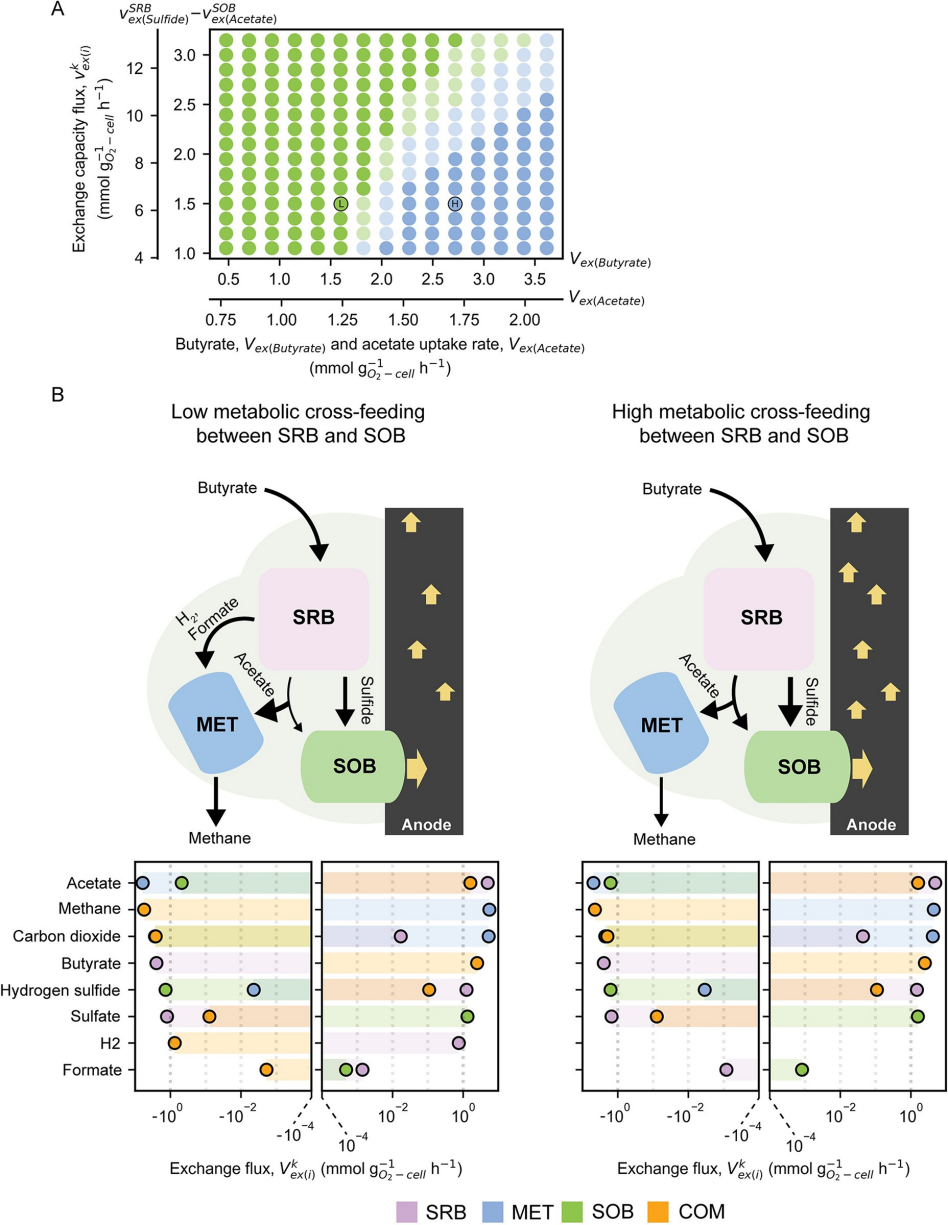
Manipulation of metabolic cross-feeding during increasing organic concentration. **A**, Dominant microbial groups under varying sulfate-reducing bacteria (SRB) and sulfide-oxidizing bacteria (SOB) exchange capacities ($v_{ex(i)}^{k}$) and organic concentrations. Dots with black edges represent the original equilibrium of the exchange capacities of SRB-SOB under the low organic loading condition (L-OLR), and high organic loading conditions (H-OLR). $v_{ex(i)}^{k}$ is an exchange capacity flux of the metabolite *i* of microbial group *k* including SRB, methanogen (MET), and SOB. **B**, Proposed microbial metabolic cross-feeding ($V_{ex(i)}^{k}$) at original and high SRB-SOB exchange capacities in H-OLR condition. $V_{ex(i)}^{k}$ is exchange reaction flux of the metabolite *i* in community compartment (COM) and the microbial group *k* including SRB, MET, and SOB. Positive (+) and negative (-) exchange fluxes indicate metabolite secretion (+) and consumption (-) for microbial compartments, while representing metabolite influx (+) and efflux (-) for COM. The exchange fluxes on x-axis are represented in an exponential scale.

**Fig 6:**
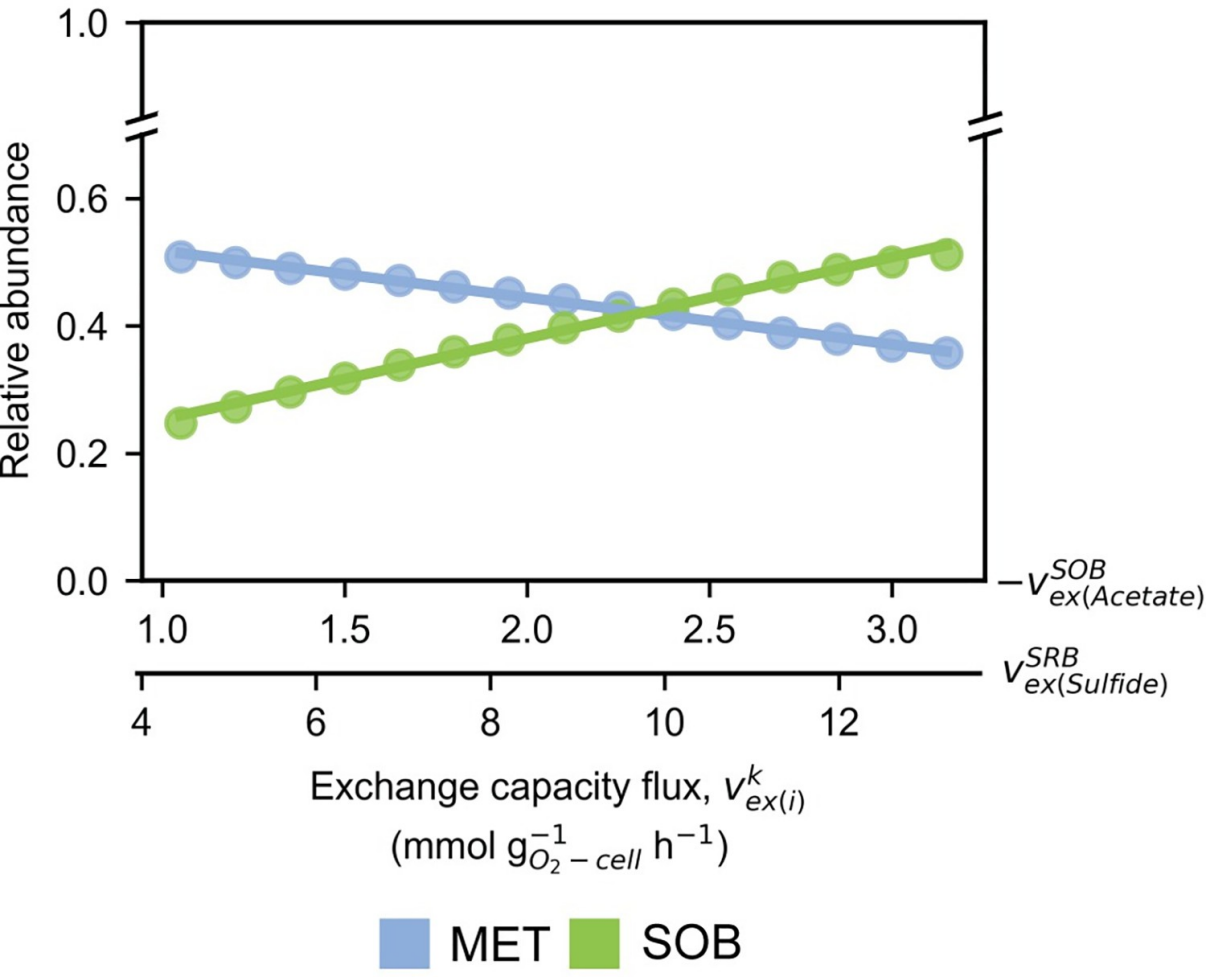
Influence of SRB-SOB exchange capacity on microbial relative abundance. Linear relationships between the exchange capacities ($v_{ex(i)}^{k}$) and relative abundances, of sulfide-oxidizing bacteria (SOB) and methanogens (MET). $v_{ex(i)}^{k}$ is an exchange capacity flux of the metabolite *i* of microbial group *k* including sulfate-reducing bacteria (SRB), MET, and SOB.

The positive correlation was observed between the abundance of SOB and current generation in a previous MFC experiment [5] (S1 Fig and S1 Table). Previous works not only proposed an association between SOB and electricity production [45–48], but also demonstrated SOB’s electricity-producing capability [49,50]. This positions SOB as a promising electroactive microbial group for MFCs. Our previous analysis suggested the significant role of SRB-SOB interactions, particularly in processes involving acetate exchange and S cycling. The relaxation of these interactions is likely to promote the growth of SOB, particularly under higher OLR conditions. Here, simulations were conducted using mmGEM to investigate how changes in the metabolic capacities of key microbial groups and organic substrate availability influence microbial interactions and community dynamics within MFCs. Specifically, the simulations explored how varying the maximum acetate consumption capacity of SOB (LB of $v_{ex(Acetate)}^{SOB}$ from -1.05 to -3.15 mmol $g_{O_{2}‐\mathrm{cell}}^{‐1}\;h^{‐1}$) and the maximum sulfide release capacity of SRB (UB of $v_{ex(Sulfide)}^{SRB}$ from 4.39 to 13.18 mmol $g_{O_{2}‐\mathrm{cell}}^{‐1}\;h^{‐1}$) could affect the system under the increased loads of organic substrates such as butyrate and acetate (Fig 5). The results showed that enhancing the metabolic capacities of SRB and SOB promotes SOB growth by strengthening their interactions. Specifically, by setting the maximum acetate consumption capacity of SOB to -3.15 mmol $g_{O_{2}‐\mathrm{cell}}^{‐1}\;h^{‐1}$ and the maximum sulfide release capacity of SRB to 13.18 mmol $g_{O_{2}‐\mathrm{cell}}^{‐1}\;h^{‐1}$, SOB proliferation was supported. Moreover, the enhanced SRB-SOB interactions enabled the microbial community to tolerate the intrusion of MET up to approximately 119% for butyrate and 64% for acetate from L-OLR condition (S4 Fig). The original equilibrium of SRB-SOB interactions at L-OLRs (LB of $v_{ex(Acetate)}^{SOB}$ = -1.50 mmol $g_{O_{2}‐\mathrm{cell}}^{‐1}\;h^{‐1}$ and UB of $v_{ex(Sulfide)}^{SRB}$ = 6.28 mmol $g_{O_{2}‐\mathrm{cell}}^{‐1}\;h^{‐1}$) could be held until the levels of butyrate and acetate rose by 28% and 15%, respectively, at which point they were disrupted and MET became predominate (S4 Fig). This resistance declined even further with weaker SRB-SOB interactions (LB of $v_{ex(Acetate)}^{SOB}$ = -1.05 mmol $g_{O_{2}‐\mathrm{cell}}^{‐1}\;h^{‐1}$ and UB of $v_{ex(Sulfide)}^{SRB}$ = 4.39 mmol $g_{O_{2}‐\mathrm{cell}}^{‐1}\;h^{‐1}$) (S4 Fig). Stronger SRB-SOB interactions notably bolster the resistance of the SOB-dominant community to elevated organic concentrations. The expansion of the transient area (lighter color) in Fig 5A indicates a slower reduction in the relative abundance of SOB and the invasion of MET. This interaction benefits SOB by enabling higher acetate and sulfide uptake from SRB, while reciprocally sharing higher sulfate at elevated OLRs (Fig 5B). The transfer of metabolites occurred incrementally without disruption, sustaining SRB metabolism through enhanced sulfate consumption, eventually hindering the production of H_2_ and formate. Consequently, MET growth is impeded due to reduced acetate uptake, leading to lower methane production. In addition, the strength of SRB-SOB interactions positively correlates with the relative abundance of SOB (Fig 6), suggesting that promoting SOB growth maintains cross-feedings, thereby preserving the community structure during organic perturbation.

### Enhancing MFC performance under high organic loading through proton concentration management

Here, mmGEM was employed to provide scenario-based circumstances of the potential metabolic intervention for sustaining MFC performance. We propose a strategy to support the growth of SOB by increasing H^+^ concentration in the environment, aiming to maintain the SOB-dominant community structure and improve MFC performance. While acetate and sulfide benefit SOB, acetate supplementation can also promote the growth of MET, whereas sulfide toxicity poses operational challenges. Previous sensitivity analysis suggests that SOB exhibit a preference for H^+^ over other microbial guilds (S2 Fig). Manipulation of H^+^ levels is also a common operational practice in wastewater treatment, and acidic conditions are detrimental to methanogens [51]. To explore this strategy further, simulations of mmGEM were performed under H-OLR conditions with varying maximum uptake rates of H^+^ at the community level (LB of $V_{ex(H^{+})}$), ranging from 4.21×10^−4^ to 4.21 mmol $g_{O_{2}‐\mathrm{cell}}^{‐1}\;h^{‐1}$ to represent the neutral to more acidic condition, assuming that all microbial guilds have ability to uptake and utilize these proton.

**Fig 7:**
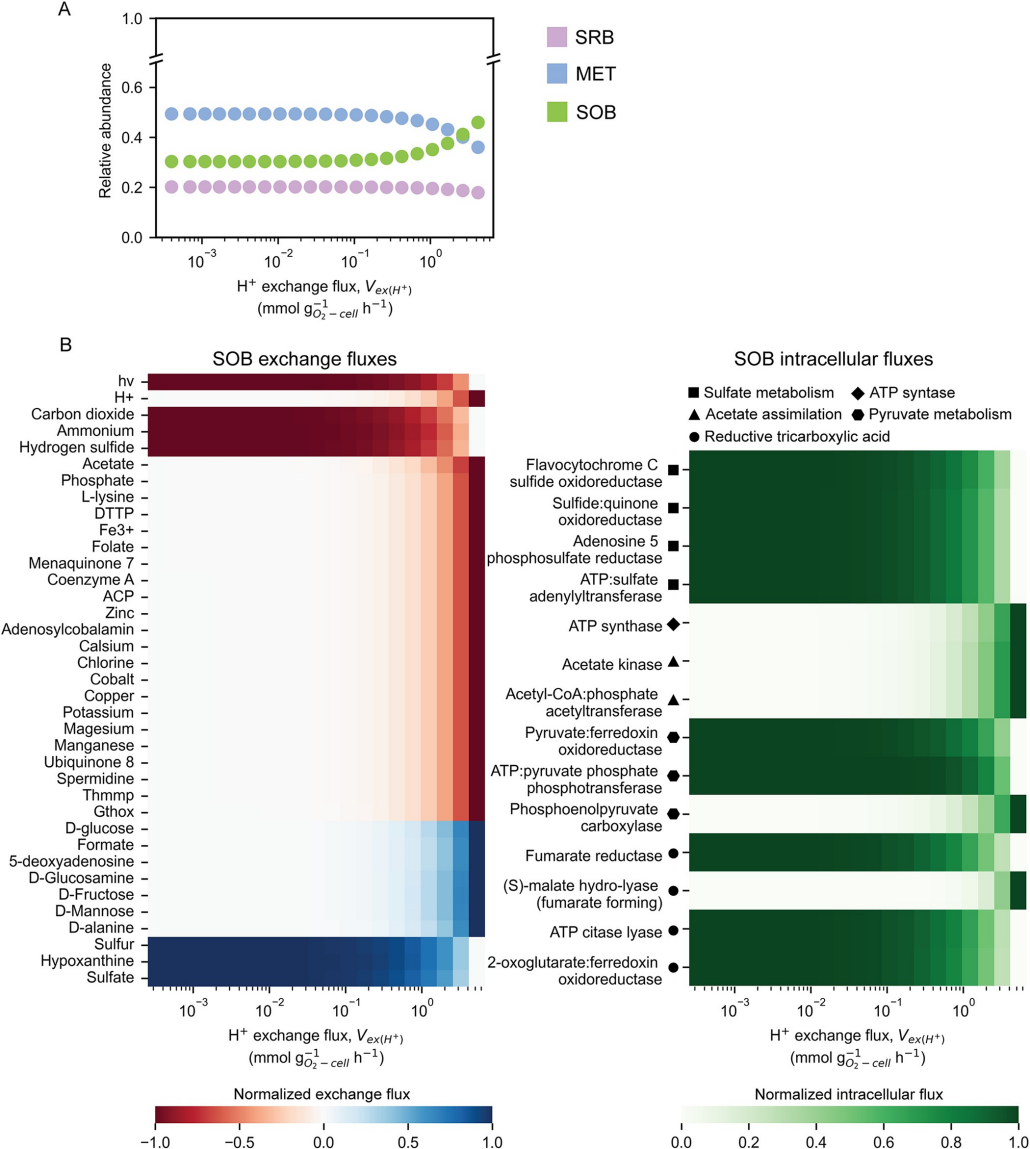
High H^+^ concentration supports growth and metabolic cross-feeding of SOB. **A**, Relative abundances of microbes including sulfate-reducing bacteria (SRB), methanogens (MET), and sulfide-oxidizing bacteria (SOB), and **B**, Intercellular cross-feeding and intracellular fluxes of SOB during increasing H^+^ concentration ($V_{ex(H^{+})}$). The simulated fluxes were normalized by min-max normalization. Positive (+) and negative (-) normalized fluxes of exchanges indicate metabolite secretion (+) and consumption (-) in SOB.

The relative abundance of SOB increased with rising H^+^ concentrations, while there was rapid drop in MET abundance, specifically at $V_{ex(H^{+})}$ = 2.66 mmol $g_{O_{2}‐\mathrm{cell}}^{‐1}\;h^{‐1}$ (Fig 7A). SOB benefited in terms of growth from higher H^+^ uptake, attributed to the ATP synthesis reaction (Fig 7B). Moreover, higher H^+^ uptake influenced elevated acetate uptake in SOB, facilitating carbon assimilation through the Pepc and Pfor (Fig 7B). The higher acetate uptake in SOB also indicated higher competitiveness for their common acetate substrate with MET. Notably, high H^+^ availability even contributed to reducing sulfide and CO_2_ uptakes from SRB, resulting in a gradual decrease in SRB growth while SOB thrived more effectively. Overall, the improved growth capacity of SOB enabled them to increase their relative abundance and dominate within the microbial community under H-OLR conditions. Consequently, the proposed scenario is expected to induce adjustments in microbial community equilibrium, thereby facilitating improvements in MFC performance.

## Discussion

The performance of MFCs in sulfide and organic-rich wastewater from the canned-pineapple industry, including electricity production and the removal of COD and sulfide, was significantly influenced by the composition and activity of the microorganisms. To investigate microbial complex interaction in MFC system, we developed a microbe-microbe interaction genome-scale metabolic model (mmGEM) using functional-based lumped compartmentalized model design to unravel the intricate metabolic cross-feeding among important microbial guilds. Since fundamental microbial functions in MFCs are driven by groups of microbial species rather than by single species, a microbial compartment in the mmGEM was modeled as a microbial guild, ideally aggregating multiple species. The mmGEM conceptual design provides effective computational simulation and interpretation with strategically retaining sufficient biological complexity to accurately represent the studied system, enabling a comprehensive understanding of the circumstances. Under this design, we demonstrated that the mmGEM could simulate an accurate microbial transition from SOB-dominant to MET-dominant during increasing OLR conditions. The approach also revealed metabolic cross-feeding mechanisms that influenced changes in microbial composition. Additionally, the function-based metabolic model effectively responded to dynamic organic substrate conditions and captured the capacity for microbial interactions. This not only provided insights into the microbial ecosystem in MFCs but also suggested a manipulative strategy for improving MFC performance. Our approach achieves a well-balanced combination of computational feasibility and realistic contextual representation with minimal data requirements. Unlike many GEM studies that rely on species-level or synthetic communities, this study offers an alternative approach that is practical for real and complex microbial systems.

The mmGEM precisely simulated microbial abundances under both L-OLR and H-OLR conditions (Fig 2A) highlighting the pivotal roles of SRB, MET, and SOB in shaping the microbial community composition in MFCs across varying OLRs. In addition, the observed microbial community adaptation is linked to metabolic cross-feeding among microbial guilds, influencing MFC performance. The persistent COD removal in both conditions can be attributed to increased acetate utilization by MET (Figs 2B and S1). This finding aligns with previous studies highlighting MET’s crucial role in COD removal, particularly in higher OLR conditions [15,16]. However, reports indicate a negative influence on electricity production in MFCs [16,52,53]. Considering the correlation between SOB and electricity generation [5,49,50], competition between MET and SOB would impact electricity generation. Simulated acetate cross-feeding highlighted this competition and the consequent dominant role of MET, resulting in lower current density in H-OLR conditions (S1 Table).

Our findings suggest that metabolic cross-feedings arise from SRB engaging in incomplete metabolism due to sulfate limitations (Fig 2). This aligns with prior reports highlighting how sulfate constraints disrupt redox balance when the organic substrate exceeds the capacity of electron acceptors, leading to the secretion of metabolic byproducts instead of completing metabolism [54,55]. The secreted metabolites, including acetate, sulfide, and H_2_ (Fig 2), probably create niches suitable for the coexistence of MET and SOB [14,56]. This coexistence leads to constant scavenging of these metabolites (Fig 2), preventing thermodynamic constraints such as the accumulation of acetate and sulfide, which could negatively impact the metabolic processes of SRB [57–59]. Under higher organic loading conditions, a smaller ratio of carbon to sulfate substrates in SRB leads to the secretion of more diverse metabolites, notably acetate, H_2_ and formate (Fig 3). These incremental secretions agree with an increased abundance of diverse methanogens (e.g., *Methanosaeta*, *Methanolinea*, and *Methanoregula*) at H-OLRs [5]. This could be explained by the necessity for greater diversity and selective function to effectively disseminate the higher free energy [60,61]. In H-OLR conditions, MET outperformed SOB in efficiently utilizing acetate (Fig 3), likely as a result of their metabolic efficiency and lower growth-dependency compared to SOB [43,62]. Although SOB can derive substantial benefits from acetate assimilation, this process relies on sulfide as an energy source [63], in line with simulation results demonstrating a correlation between the decline in S cycling efficiency and reductions in both acetate assimilation and biomass biosynthesis (Figs 2 and 3).

The simple exchange of catabolic metabolites, involving S cycling and acetate assimilation between SRB and SOB, significantly determined metabolic cross-feeding and subsequent shifts in community composition (Fig 4). The original equilibrium state of the microbial community demonstrated resilience against an organic increment of approximately 28% before transitioning into a community structure more conducive to SOB growth and activity (Fig 5). These findings align with those of Sriwichai et al. (2024), who noted sustained high current generation in another MFC operating condition even when the rate of organic loading into the MFC chambers remained constant. This sustained high current generation at a constant OLR is likely due to the sustained growth and metabolic activity of SOB over time [5]. Our findings demonstrate a strong SRB-SOB interaction, which facilitated consistent acetate cross-feeding, preventing the development of a niche for MET. Similarly to Dolinšek et al. (2022), our study underscores how the initial microbial composition can gradually shape metabolic cross-feeding and community structure by gradually influencing environmental conditions over time [64]. This suggests that augmenting the initial abundance of SOB can strengthen their bonds with SRB (Fig 6), thereby enhancing the community’s resilience against fluctuations in organic concentrations.

While some studies have suggested the detrimental effect of extreme acidity on MFC performance, others have reported improved electrical production in an acidified anodic environment [65,66]. Our simulations highlighted the differential responses of microbial guilds to acidity within the MFC environment, with SOB showing a favorable response in terms of growth and ATP generation compared to SRB and MET (Fig 7). Previous research has highlighted the species-specific nature of microbial responses to acidity. For example, it was observed that *Pseudomonas putida* showed an increase in cellular ATP under high environmental H^+^ conditions, while *Staphylococcus epidermidis* stabilized ATP synthesis in the same acidic conditions [21]. The increased growth of SOB in response to H^+^ supplementation in the MFC system is associated with an improvement in cross-feeding interactions with SRB, which led to a reduction in substrates typically utilized by MET, thereby limiting their growth and activity (Fig 7B). The fact that acetoclastic methanogens do not thrive in acidic environments provides additional evidence to support the idea that the perturbation caused by the increase in H^+^ ions can significantly impact the microbial dynamics within the system [51]. Here, we propose that a temporal H^+^ perturbation could be used to exploit the SRB-SOB interactions to suppress the competitiveness of MET. The MFC microbiome can then recover from a collapse caused by low pH conditions once a neutral pH is restored [20]. Further investigation is, however, required to determine effective conditions for inducing H^+^ perturbation in MFCs.

This study highlights the potential of MFC technology to operate sustainably in industrial settings, even amidst fluctuating conditions. By addressing critical challenges in its implementation, our simulated results indicate that controlled perturbations introduced to the microbial community within MFC systems can enhance microbial structure and improve MFC performance under high organic loading conditions. Furthermore, the mmGEM proves to be an effective tool for studying complex interactions within MFC systems. Despite requiring minimal data for its construction and simulations, the model provides detailed insights into metabolic processes and composition changes within the microbial community. In essence, mmGEM validity is leveled by the extent of comprehensive knowledge about the metabolic properties of microorganisms (i.e. as to define: a choice selection of metabolic-specific microbial guilds) and the key metabolic processes of the system (i.e. as to define a choice selection of essential metabolic functions to achieve the system’s outcome) that are taken into account for modeling. In this case, the mmGEM has demonstrated its reliability by effectively responding to the increasing OLR covering the scope of the studied conditions (Fig 4). Additionally, it remains robust across various ranges of parameterized cross-feeding reactions (S2 Text). Further expanding the model’s scope to cover a wider range of substrates and microbial species, including fermentative bacteria, would better simulate processes influencing electricity generation in MFCs and provide a more comprehensive representation of chemical balances within the system [11,67]. While the mmGEM relies on a constant cell yield assumption (*Y*_*cell mass*/*substrate*_ = 0.15 ${\mathrm{mg}}_{O_{2}‐\mathrm{cell}}\;{\mathrm{mg}}_{O_{2}‐\mathrm{substrate}}^{‐1}$) derived from a previous MFC study using acetate as a substrate [68], we recommend reevaluating this assumption to account for potential variations in cell yield under different substrate conditions. Despite falling within the typical range observed in anaerobic wastewater treatments and MFCs (0.04–0.25 ${\mathrm{mg}}_{O_{2}‐\mathrm{cell}}\;{\mathrm{mg}}_{O_{2}‐\mathrm{substrate}}^{‐1}$) [69,70], such reevaluation could improve the accuracy of MFC modeling.

## Conclusions

This work highlights the significant role of metabolic cross-feeding in shaping microbial composition and MFC performance under varying OLRs. Through functional-based lumped compartmentalized modeling, the mmGEM facilitated a comprehensive study of the intricate dynamic metabolic interactions within microbial communities in MFCs, ultimately enhancing our understanding of microbial ecology and facilitating the optimization of MFC performance for various biotechnological applications. Here, it presents a detailed description of the transition from an SOB-dominant community at L-OLRs to a MET-dominant community at H-OLRs, as the MFC performance declines. This transition can be attributed to dynamic interactions among microbial groups in the system. Our findings unveiled various cross-feeding mechanisms. Specifically, we observed a deterioration in SRB-SOB interactions, including S cycling and acetate cross-feeding. Additionally, we identified efficient acetate consumption by MET. These interactions collectively led to changes in the composition and structure of the microbial community within the MFC system, finally impacting performance. We propose a manipulation strategy to enhance MFC performance by sustaining the dominance of SOB under H-OLR conditions. By increasing the amount of proton (H^+^) in the MFC environment, we simulate the growth of SOB, enhance ATP production, strengthen SRB-SOB interactions, and boost MFC performance. The anticipated outcomes offered a hopeful prospect for the long-term functioning of MFCs, illuminating potential future applications within the industrial domain.

## Materials and methods

### Construction of mmGEM

To investigate metabolic cross-feeding interactions among microbial guilds within MFCs, a functional-based lumped compartmentalized structural model called mmGEM (microbe-microbe interaction genome-scale metabolic model; S1 File) was designed such that each compartment represented the metabolism of individual microbial guilds aggregated within a fictitious community compartment (COM) [35]. The COM serves as an extracellular space for metabolite sharing among microbial guilds, facilitating collaborative metabolic processes crucial for community stability and functions.

The mmGEM was constructed based on a comprehensive set of data (see S1 Text), including the predominant microbial guilds in MFCs derived by 16S-rRNA analysis under the study conditions (S1 Table), the corresponding MFC performance metrics (S1 Fig), the biochemical composition of influent and effluent wastewater, and existing knowledge of microbial guild characteristics [13,42,43]. The mmGEM comprises three compartments representing different microbial guilds in MFCs: SRB are represented by the genus *Acinetobacter*, MET by *Methanothrix* (the genus was called *Methnosaeta* [71]), and SOB by *Chlorobaculum*, with one common spatial compartment (COM) to facilitate metabolite exchange. Specifically, *A*. *calcoaceticus* [72,73], *M*. *soehngenii* [43,74], *and C*. *limnaeum* [75] were chosen to represent each guild based on shotgun metagenome analysis of MFCs under corresponding conditions [5] and based on their unique metabolic functions. The metabolic interactions within the MFC microbial community are underpinned by several foundational assumptions. First, it is assumed that microbial species interact freely with each other based on their unique metabolic capabilities, fostering a network of nutrient exchange within the community. Additionally, the utilization of nutrients is assumed to maintain a steady-state condition, implying a constant rate of nutrient consumption and metabolism over time. SRB are expected to predominantly utilize organic compounds as carbon and energy sources, with sulfate respiration leading to the production of sulfide as a byproduct [13]. MET are assumed to primarily engage in acetoclastic methanogenesis, converting acetate into methane [14]. SOB are assumed to exhibit a hetero-autotrophic lifestyle, meaning they can utilize both organic compounds (acetate and pyruvate) and CO_2_ for growth and energy production, as well as fix atmospheric CO_2_ through photosynthesis and sulfur metabolism [56,62,76]. Additionally, SOB are assumed to act as electrogenic bacteria within MFCs. This assumption is based on a reported positive correlation between their abundance in MFCs and electricity generation [5], as well as previous reports on SOB’s capacity to perform extracellular electron transfer with anode electrode [49,50].

The GEM for each microbial guild was constructed by leveraging the genome information of the representative species. The draft models for MET and SOB were sourced from ModelSEED [77], whereas the SRB model was obtained from the AGORA database [78]. The draft models were subsequently refined, focusing on biomass biosynthesis reactions and representative pathways. To enhance accuracy, biomass and macromolecule biosynthesis equations were refined using information from GEMs of closely related species [79,80], relevant literature [81,82], and estimations based on the species’ genomes [83]. The general characteristics of the microbial guilds were refined using information from biochemical databases such as KEGG [84], MetaCyc [85], and BiGG [86], as well as existing literature, particularly studies on dissimilatory sulfate reduction by SRB [87,88], acetoclastic methanogenesis by MET [89,90], and sulfur metabolism, photosynthesis, and CO_2_ fixation by SOB [42,91]. The individual microbial GEMs were finally combined using the Microbiome Modeling Toolbox [92], allowing for the representation of metabolic interactions and cross-feeding relationships among the different microbial guilds within the MFC system. The resulting mmGEM underwent general curation steps, including mass balance checks and elimination of active thermodynamic infeasible loops, to enhance its reliability and accuracy.

### Model simulation

The simulation of metabolic cross-feeding using the mmGEM was conducted under L-OLR conditions at 216 days after initiation of the MFC operation (DAO) (S1 Fig). The concentrations of the wastewater components, including organic compounds (measured as chemical oxygen demand; COD), sulfate, and sulfide, were calculated. These concentrations were used to determine condition-specific rates of metabolite influxes and effluxes (mmol $g_{O_{2}‐\mathrm{cell}}^{‐1}\;h^{‐1}$). It was assumed that the total organic compounds present in the wastewater (COD) mainly consisted of VFAs like butyrate and acetate. These VFAs were estimated to constitute up to 63.12% of the whole organic compounds in the wastewater. It was further assumed that these VFAs were utilized by EB [3] and other selected microbial guilds within the community [13,42,43]. The specific rates of butyrate, acetate, sulfate, and sulfide were determined based on their mass concentration and the estimated cell mass of SRB, MET, and SOB. The mass concentrations of butyrate and acetate from the organic measurement obtained through COD analysis, particularly from the measured VFAs level at 286 DAOs. It was assumed that all organic compounds were completely oxidized, and molar proportions of VFAs to non-VFAs and those between the VFA species were constant throughout the system’s operation. The cell mass of SRB, MET, and SOB was estimated from the cell yield (*Y*_*cell mass*/*substrate*_ = 0.15 ${\mathrm{mg}}_{O_{2}‐\mathrm{cell}}\;{\mathrm{mg}}_{O_{2}‐\mathrm{substrate}}^{‐1}$), representing the ratio of cell mass produced to the substrate consumed. This estimation was anchored in a previous study under a similar MFC environment, where the cell yield was determined to be constant across operational states [68]. Data on microbial relative abundance from a canned-pineapple industrial MFC system [5] was incorporated into the estimation process. In addition, a minimal set of exchange metabolites at COM, such as photons, water, and phosphate, were allowed to freely exchange due to model’s requirements. All constraints in mmGEM are detailed in S2 Text.

The mmGEM simulation aimed to maximize microbial community growth (*μ*) under steady-state conditions using SteadyCom [37], a microbial community GEM optimization framework. SteadyCom simulated microbial relative abundances by assuming constant interactions and equal growth rates between microbes, while properly weighting metabolite exchange based on the relative abundance. The optimization equation below (Eq 1) encapsulates this process.

max μ

subject to

$$\left[\begin{array}{l} \sum_{j\in J^{k}}S_{ij}^{k}V_{j}^{k}=0,\;\forall i\in I^{k}\\ {LB}_{j}^{k}X^{k}\leq V_{j}^{k}\leq UB_{j}^{k}X^{k},\;\forall j\in J^{k}\\ V_{biomass}^{k}=X^{k}\mu \\ V_{j}^{k}=v_{j}^{k}X^{k}\\ X^{k}\geq 0 \end{array}\right]\;\forall k\in K$$ (1)

$$\begin{array}{l} u_{i}^{c}—e_{i}^{c}+\sum_{k\in K}V_{ex(i)}^{k}=0,\;\forall i\in I^{com}\\ \sum_{k\in K}X^{k}=X_{0}\\ u_{i}^{c},\;e_{i}^{c}\geq 0,\;\forall i\in I^{com} \end{array}$$

where *μ* is the community growth rate (h^-1^); $S_{ij}^{k}$ is the stoichiometric coefficient of metabolite *i* in reaction *j* within organism *k*; $V_{j}^{k}$ is the aggregated flux (mmol $g_{O_{2}‐\mathrm{cell}}^{‐1}\;h^{‐1}$) of reaction *j* within organism *k*. This flux value results from $v_{j}^{k}\;(\mathrm{mmol}\;g_{O_{2}‐\mathrm{cell}}^{‐1}\;h^{‐1})$ multiplied by *X^k^*; $v_{j}^{k}$ is the capacity of organism *k* to produce flux for reaction *j* per unit of its relative abundance (*X*^*k*^); *X*_0_ is the total relative abundance of the microbial community and is typically set to 1 by default; ${LB}_{j}^{k}$ and $UB_{j}^{k}$ represent the lower and upper boundaries of $v_{j}^{k}\;(\mathrm{mmol}\;g_{O_{2}‐\mathrm{cell}}^{‐1}\;h^{‐1});\;V_{ex(i)}^{k}$ is the exchange reaction of metabolite *i* within organism *k* between the microbial and community compartments; and $u_{i}^{c}$ and $e_{i}^{c}$ are the uptake and export reactions, respectively, of metabolite *i* between the community compartment and the outside environment.

The simulation was carried out using the COBRA toolbox (version 3.0.4) [93] within the MATLAB environment (The Math Works, version R2022b). The computation was performed on a laptop computer equipped with a Dual-Core Intel Core i5 processor and 8 GB of memory. The mmGEM, along with the condition-specific data and the script for running mmGEM on SteadyCom, is available in the GitHub repository KMUTT-CASB/mmGEM (https://github.com/KMUTT-CASB/mmGEM).

### Model verification

The cross-feeding interactions among microbial guilds, as modeled using mmGEM under L-OLR conditions, were evaluated for their applicability in H-OLR conditions (S1 Fig). Specifically, the cross-feedings modeled at L-OLRs were examined to determine if they could be extrapolated to predict microbial interactions and structure in H-OLR conditions.

### Comparison of metabolic fluxes in L-OLR and H-OLR

To compare metabolic activity between L-OLR and H-OLR conditions, we contrasted the predicted fluxes, including intracellular fluxes ($V_{j}^{k}$) and exchanges fluxes ($V_{ex(i)}^{k}$), using flux fold change (FFC, Eq 2). A reaction flux lower than 1×10^−6^ mmol $g_{O_{2}‐\mathrm{cell}}^{‐1}\;h^{‐1}$ was assumed to be zero.


$$Flux\;fold\;change={log}_{2}\left(\frac{V_{\left(H-OLR\right)}^{k}}{V_{\left(L-OLR\right)}^{k}}\right)$$ (2)

### Model sensitivity analysis

Sensitivity analyses were conducted to investigate the effect of cross-feeding on microbial community composition under dynamic OLRs and metabolic manipulations aimed at sustaining SOB growth under H-OLR conditions. The mmGEM was used initially to study metabolic cross-feeding under varying organic concentrations, focusing on the uptake rates of butyrate (*V*_*ex*(*Butyrate*)_) and acetate (*V*_*ex*(*Acetate*)_). The rates of butyrate and acetate uptake into COM were linearly increased while other constraints and conditions within the metabolic model were kept constant to maintain consistency with L-OLR conditions. A series of absolute metabolite exchange fluxes ($V_{ex(i)}^{k}$), denoted as ${SF}_{ex(i)}^{k}$ where ${SF}_{ex(i)}^{k}=\left\{{\left|V_{ex(i)}^{k}\right|}_{1},{\left|V_{ex(i)}^{k}\right|}_{2},{\left|V_{ex(i)}^{k}\right|}_{3},\ldots ,{\left|V_{ex(i)n}^{k}\right|}_{n}\right\}$, were scaled using min-max normalization (Eq 3) to capture cross-feeding behavior. Following normalization, the original flux direction symbols (+ or -) were reinstated to indicate flux directions. Investigation of the exchange capacity flux ($v_{ex(i)}^{k}$) was also conducted using the same protocol.


$$Normalized\;flux=\frac{{SF}_{ex(i)n}^{k}-min\;{SF}_{ex(i)}^{k}}{\max\;{SF}_{ex(i)}^{k}-min\;{SF}_{ex(i)}^{k}}$$ (3)

The mmGEM was further expanded to investigate how the sulfide production capacity of SRB (UB of $v_{ex(Sulfide)}^{SRB}$) and acetate consumption capacity of SOB (LB of $v_{ex(Acetate)}^{SOB}$) influence microbial community composition. A scenario-based analysis was conducted to explore potential improvements in SOB growth under H-OLR conditions. This involved exponentially increasing the maximum rate of H^+^ exchange between the outside environment and COM (LB of $V_{ex(H^{+})}$).

## Supporting information

S1 Text Conceptual design of mmGEM. (DOCX)

S2 Text Constraints and Parameterization of mmGEM. (DOCX)

S1 Table Relative abundances of microbial groups. (XLSX)

S2 Table Components of mmGEM. (XLSX)

S3 Table Simulation of metabolic flux conversion in microbial community. (XLSX)

S4 Table List of acronyms. (XLSX)

S1 Fig MFC performance and condition-specific data under L-OLR and H-OLR conditions. **A**, Performance of MFC from previous experiments under low organic loading (L-OLR) and high organic loading (H-OLR) conditions [5]. **B**, The model input flux (*V*_*ex*(*i*)_) for representing the MFC specific conditions in L-OLR and H-OLR. *V*_*ex*(*i*)_ is an exchange reaction flux of the metabolite *i* in community compartment (COM) Positive (+) and negative (-) exchange fluxes indicate metabolite influx (+) and efflux (-) for COM.
(TIF)

S2 FigParameterization of H^+^ exchange capacity.**A**, Average percent error of microbial relative abundances, including sulfate-reducing bacteria (SRB), methanogens (MET), and sulfide-oxidizing bacteria (SOB), between model simulation and experimental data in the low organic loading condition (L-OLR) and **B**, H^+^ exchange flux ($V_{ex(H^{+})}^{k}$) while perturbing H^+^ exchange capacities of SRB ($v_{ex(H^{+})}^{SRB}$) and MET $(v_{ex(H^{+})}^{MET}).\;V_{ex(H^{+})}^{k}$ is an exchange reaction flux of H^+^ in community compartment (COM) and the microbial group *k* including SRB, MET, and SOB. Positive (+) and negative (-) exchange fluxes indicate metabolite secretion (+) and consumption (-) for microbial compartments, while representing metabolite influx (+) and efflux (-) for COM.(TIF)

S3 FigNormalized exchange fluxes under increasing organic concentration.Normalized exchange fluxes ($V_{ex(i)}^{k}$) of sulfate-reducing bacteria (SRB), methanogens (MET), and sulfide-oxidizing bacteria (SOB) under increasing organic concentration. The fluxes of an exchange reaction across the increasing organic concentration were normalized by min-max normalization. The first and second phases are separated by a dashed-blue line. The first phase is defined between 0.48 and 1.26 mmol $g_{O_{2}‐\mathrm{cell}}^{‐1}\;h^{‐1}$ for butyrate uptake rate and 0.77 to 1.10 mmol $g_{O_{2}‐\mathrm{cell}}^{‐1}\;h^{‐1}$ for acetate uptake rate. The second phase ranges from 1.37 to 3.61 and 1.15 to 2.09 mmol $g_{O_{2}‐\mathrm{cell}}^{‐1}\;h^{‐1}$ for butyrate and acetate uptake rates, respectively. Dashed-grey lines represent points of the organic concentration, equal to low organic loading condition (L-OLR) and high organic loading conditions (H-OLR). $v_{ex(i)}^{k}$ is exchange capacity flux of the metabolite *i* of microbial group *k* including SRB, MET, and SOB. Positive (+) and negative (-) normalized exchange capacity fluxes indicate metabolite secretion (+) and consumption (-) for microbial compartments.(TIF)

S4 FigSimulated microbial relative abundances under increasing organic concentration at different SRB-SOB exchange capacities.The growth patterns of sulfate-reducing bacteria (SRB), methanogens (MET), and sulfide-oxidizing bacteria (SOB), are from (A) high, (B) original, and (C) low exchange capacities of S cycling ($v_{ex(Sulfide)}^{SRB}$) and acetate cross-feeding ($v_{ex(Acetate)}^{SOB}$) between SRB and SOB.(TIF)

S1 File mmGEM model. (SBML)
