# Supplementary material for: Metabolic cross-feeding interactions modulate the dynamic community structure in microbial fuel cell under variable organic loading wastewaters
Source: PLoS Comput Biol. 2024 Oct 17;20(10):e1012533. doi: 10.1371/journal.pcbi.1012533 (PMC11521316; doi:10.1371/journal.pcbi.1012533)
Supplement: S1 Text — (DOCX) [file pcbi.1012533.s001.docx]

**S1 Text: Design of the mmGEM**

Since a wide variety of species are present in microbial communities, it is well recognized that these interactions are extremely complex. Its symbiosis's complexity is always on the edge of, or frequently beyond, what can be measured. To provide insight into the system, a computational method has been suggested to model the potential situations that could underlie the quantifiable fingerprints seen throughout the trials. The approach has been shown powerful and well facilitates an in-depth study of complex microbial interaction in various contexts [1–3], though inevitably accounting for certain simplifying assumptions. The simplification makes analytical computation viable but brings with it a restriction on the validity range of predictions. In this study, the microbe-microbe interaction genome-scale metabolic model (mmGEM) was conceptually created to investigate a metabolic cross-feeding situation that microbial cohorts engage and shape the community network in microbial fuel cells (MFCs) in the canned-pine apple industry. Supposed a microbial cohort refers to microbial species sharing metabolic characteristics, thereby acting similarly in a process of MFC organic conversion. To minimize complexity arising from typical incomplete microbiota data, the mmGEM was modeled using microbial guilds specific to metabolism. This simplified conceptual design enables the inclusion of more microbial species in the study while limiting the number of microbial compartments in the community model. Additionally, a larger community can account for a larger proportion of the nutrients present in the real environment. This reduction in complexity makes metabolic modeling more representative of actual conditions, particularly in complex systems such as in wastewater treatment. Despite model simplicity, the mmGEM demonstrated in this study that changes in interactions at the level of the microbial guild are sufficient to explain the transition of community structure in this wastewater treatment MFCs.

For mmGEM conceptual design and simulation, we employed the microbiome analysis and MFC contextual data from experiment of Sriwichai et al. (2024) [4]. The process of choosing the microbial compartment in mmGEM involved two steps: (1) determining the important microbial guilds by examining their correlation with the conditions and performance of MFC wastewater; and (2) choosing representative species for each guild to serve as genomic templates in the model reconstruction phase. First, the key microbial guilds were selected based on biochemical information from wastewater, microbial relative abundance, and the performance of the MFC system. In this MFC system, the wastewater from the canned pineapple industry contained organic compounds and sulfur compounds (sulfate and sulfide), which influenced the structure of the microbial community in these MFCs. The presence of sulfate-reducing bacteria (SRB) and sulfide-oxidizing bacteria (SOB) corresponded to the sulfur compounds, while methanogens (MET) were associated with high levels of volatile fatty acids (VFAs). These three microbial guilds cover the main function of carbon and sulfur metabolisms, which are the major contaminants in wastewater. In addition, the relative abundances of SRB, MET, and SOB were also associated with the MFC’s performance, i.e., chemical oxygen demand (COD) removal, sulfide removal, and current generation, particularly under increasing organic loading rate (OLR) (S1 Fig and S1 Table).

Second, each microbial guild's representative species were chosen for the subsequent model reconstruction. As the mmGEM was designed to investigate the complex environmental transitions in wastewater treatment MFCs, the simplified microbial guilds were decided based on three main criteria: (1) demonstrates their relative abundances highly associated with the OLR alteration, for selecting microbial genera; (2) a highly abundant microbial cohort overall; and (3) possess metabolic functions relevant to organic conversion in MFC with high sulfide wastewater from the canned pineapple industry, for selecting microbial species. Representative genera were chosen based on microbial profiles from 16S rRNA analysis during “low season” (L-OLR) and “high season” (H-OLR). The genus selected as a representative was the one that showed the greatest shift in absolute relative abundance within microbial guilds in response to the increasing OLR conditions. This significant change in microbial abundance indicated that these genera were highly affected by environmental changes and closely correlated with the MFC’s performance. S1 Text Table 1 showed that *Acinetobacter*, *Methanothrix*, and *Chlorobaculum* largely changed in relative abundance upon the increased OLRs comparing to others in the guild, thus were chosen as the representative genera for SRB, MET, and SOB, respectively.

**S1 Text Table 1** Changes in microbial relative abundance upon the shift of OLRs during the transition from low (L-OLR) to high (H-OLR) production season.

| **Microbial guilds** | **Microbial genera** | **Relative abundance (%)** | | **Difference of the relative abundance in L-OLR and H-OLR conditions (%)** |
| --- | --- | --- | --- | --- |
|  |  | **L-OLR** | **H-OLR** |  |
| SRB | *Desulfomicrobium* | 0.86 | 0.34 | 0.51 |
|  | *Desulfovibrio* | 0.73 | 0.45 | 0.27 |
|  | *Acinetobacter* | 0.66 | 3.06 | 2.40 |
| MET | *Methanolinea* | 1.11 | 2.12 | 1.01 |
|  | *Methanoregula* | 0.38 | 2.00 | 1.63 |
|  | *Methanothrix* | 2.52 | 7.92 | 5.40 |
| SOB | *Chlorobaculum* | 4.25 | 0.79 | 3.46 |
|  | *Chlorobium* | 2.69 | 0.66 | 2.03 |
|  | *Arcobacter* | 0.01 | 0.72 | 0.72 |
|  | *Sulfurovum* | 0.07 | 0.97 | 0.91 |
|  | *Spirillum* | 0.00 | 3.14 | 3.14 |
|  | *Pseudomonas* | 0.82 | 0.23 | 0.58 |

For each selected microbial genera, the representative species were chosen based on the shotgun metagenome collected at 260 days after operation (DAO) in the same MFC chamber (unpublished data). It was assumed that while the relative abundance of microbial species within the MFC can fluctuate, these species consistently exist throughout the operation period. The representative species were chosen based on their high abundance and functional roles in their associated guilds, as determined by a literature review. S1 Text Table 2 shows the abundance (reads count) for each species within the representative genera. For SRB, *Acinetobacter calcoaceticus* was chosen as the representative species. Although its abundance was not the highest within its genus, *A. calcoaceticus* is only one reported to contain sulfate-reducing capacity among other presented in this genus [5]. For MET and SOB, *Methanothrix soehngenii* and *Chlorobaculum limnaeum* were selected based on their highest abundance and relevant functions within their microbial guilds [6,7].

**S1 Text Table 2** Abundance of microbial species presented in the representative genus that was observed in microbial fuel cell at 260 days after operation.

| **Microbial guild** | **Microbial species** | **Abundance** |
| --- | --- | --- |
| SRB | *Acinetobacter sp. ACNIH2* | 14810 |
|  | *Acinetobacter pittii* | 10507 |
|  | *Acinetobacter nosocomialis* | 8062 |
|  | *Acinetobacter calcoaceticus* | 7399 |
|  | *Acinetobacter lactucae* | 7336 |
|  | *Acinetobacter indicus* | 6796 |
|  | *Acinetobacter sp. SWBY1* | 6597 |
|  | *Acinetobacter sp. LoGeW2-3* | 6185 |
|  | *Acinetobacter sp. ACNIH1* | 5642 |
|  | *Acinetobacter sp. NCu2D-2* | 4949 |
|  | *Acinetobacter sp. TGL-Y2* | 4410 |
|  | *Acinetobacter johnsonii* | 3207 |
|  | *Acinetobacter schindleri* | 3112 |
|  | *Acinetobacter radioresistens* | 2898 |
|  | *Acinetobacter equi* | 2743 |
|  | *Acinetobacter bereziniae* | 2601 |
|  | *Acinetobacter haemolyticus* | 2456 |
|  | *Acinetobacter junii* | 2316 |
|  | *Acinetobacter sp. ADP1* | 2144 |
|  | *Acinetobacter soli* | 1771 |
|  | *Acinetobacter sp. TTH0-4* | 1485 |
|  | *Acinetobacter larvae* | 1251 |
|  | *Acinetobacter calcoaceticus/baumannii complex* | 1249 |
|  | *Acinetobacter baumannii* | 938 |
|  | *Acinetobacter oleivorans* | 797 |
| MET | *Methanothrix soehngenii* | 242982 |
| SOB | *Chlorobaculum limnaeum* | 66537 |
|  | *Chlorobaculum tepidum* | 12496 |
|  | *Chlorobaculum parvum* | 3995 |

The mmGEM ultimately comprised three microbial guilds: SRB, MET, and SOB, represented by *A. calcoaceticus*, *M. soehngenii*, and *C. limnaeum*, respectively. A set of annotated biochemical equations from these microbial guilds was used to simulate substrate and product conversions with linear programming optimization. For example, serial conversion from organic substrates from the environment through complicated metabolic cross-feeding to cellular metabolisms and finally to their biomasses. The simplified equations of main organic compounds and microbial growth were listed in Equation A-E. Equation A-B shows organic compounds, butyrate, and acetate that can be influenced by the environment and interact with microbes. At the same time, these microbial growth rates (µ) also depend on the organic substrates while competing or sharing metabolite with others (Eq C-E).

$\left[ Butyrate \right]=f(\mu^{SRB}, environment)$ (A)

$\left[ Acetate \right]=f(\mu^{SRB},\mu^{MET},\mu^{SOB}, environment)$ (B)

$\mu^{SRB}=f\left( \left[ Butyrate \right], \mu^{MET},\mu^{SOB} \right)$ (C)

$\mu^{MET}=f(\left[ Acetate \right],\mu^{SRB}, \mu^{SOB})$ (D)

$\mu^{SOB}=f(\left[ Acetate \right],\mu^{SRB}, \mu^{MET})$ (E)

**References**

1. Zhuang K, Izallalen M, Mouser P, Richter H, Risso C, Mahadevan R, et al. Genome-scale dynamic modeling of the competition between Rhodoferax and Geobacter in anoxic subsurface environments. ISME Journal. 2011;5: 305–316. doi:10.1038/ismej.2010.117

2. Islam MM, Le T, Daggumati SR, Saha R. Investigation of microbial community interactions between Lake Washington methanotrophs using genome-scale metabolic modeling. PeerJ. 2020;2020. doi:10.7717/peerj.9464

3. Basile A, Campanaro S, Kovalovszki A, Zampieri G, Rossi A, Angelidaki I, et al. Revealing metabolic mechanisms of interaction in the anaerobic digestion microbiome by flux balance analysis. Metab Eng. 2020;62: 138–149. doi:10.1016/j.ymben.2020.08.013

4. Sriwichai N, Sangcharoen R, Saithong T, Simpson D, Goryanin I, Boonapatcharoen N, et al. Optimization of microbial fuel cell performance application to high sulfide industrial wastewater treatment by modulating microbial function. PLoS One. 2024;19. doi:https://doi.org/10.1371/journal.pone.0305673

5. Han Z, Zhao Y, Yan H, Zhao H, Han M, Sun B, et al. Struvite Precipitation Induced by a Novel Sulfate-Reducing Bacterium Acinetobacter calcoaceticus SRB4 Isolated from River Sediment. Geomicrobiol J. 2015;32: 868–877. doi:10.1080/01490451.2015.1016247

6. Tank M, Liu Z, Frigaard NU, Tomsho LP, Schuster SC, Bryant DA. Complete genome sequence of the photoautotrophic and bacteriochlorophyll e-synthesizing green sulfur bacterium Chlorobaculum limnaeum DSM 1677T. Genome Announc. 2017;5. doi:10.1128/genomeA.00529-17

7. Huser BA, Wuhrmann K, Zehnder AJB, Zehnder AJB. Methanothrix soehngenii gen. nov. sp. nov., a New Acetotrophic Non-hydrogen-oxidizing Methane Bacterium. Arch Microbiol. 1982;132: 1–9.
