# Supplementary material for: Metabolic cross-feeding interactions modulate the dynamic community structure in microbial fuel cell under variable organic loading wastewaters
Source: PLoS Comput Biol. 2024 Oct 17;20(10):e1012533. doi: 10.1371/journal.pcbi.1012533 (PMC11521316; doi:10.1371/journal.pcbi.1012533)
Supplement: S2 Text — (DOCX) [file pcbi.1012533.s002.docx]

**S2 Text: Constraints and parameterization of mmGEM**

**Constraints of mmGEM**

In the context of the microbe-microbe interaction metabolic model (mmGEM), the constraints at the community compartment (COM) refer to the exchange interactions between the community and the outside environment. These constraints can vary depending on the specific conditions: low organic loading rate (L-OLR) and high organic loading rate (H-OLR). Additionally, exchange reactions that are deemed more stable by the conditions are subject to be constant limitations during the conditional simulation.

For the condition-specific constraints, as described in the Materials and Methods section, the main metabolic exchanges within the microbial community in the L-OLR and H-OLR conditions were butyrate, acetate, sulfate, and sulfide as shown in S2 Text Table 1. The determination of these constraints was based on the following main assumptions:

- - Total organic compounds in the wastewater, measured as chemical oxygen demand (COD), consisted primarily of volatile fatty acids (VFAs), accounting for approximately 63.12% of the total organic compounds [1]. The molar proportions of these VFAs to non-VFAs, as well as the ratios between different VFA species, remained constant throughout the system's operation. These organic compounds were completely oxidized in the MFC environment.
  - Cell yield was employed to convert mass of substrate utilized in MFC to microbial cell biomass ($Y_{cell mass/substrate}$). The mmGEM was simulated based on the $Y_{cell mass/substrate}=$0.15 $\text{mg}_{\text{O}_{\text{2}}\text{-cell}}\text{ }\text{mg}_{\text{O}_{\text{2}}\text{-substrate}}^{\text{-1}}$, as reported in the previous MFC study under a VFA-rich environment [2].
  - For estimation of maximum H^+^ uptake (S2 Text Table 1), it was assumed that the pH stayed neutral in both the L-OLR and H-OLR circumstances. Proton concentration ([H^+^]) was based on $pH$ equation, $pH=-\log\left( \left[ H^{+} \right] \right)$.

These assumptions were critical in accurately defining the constraints for the L-OLR and H-OLR conditions, enabling a comprehensive analysis of the community's interactions with its environment under varying organic loading rates. Under these assumptions, particularly the constant mass conversion ($Y_{cell mass/substrate}$), the mmGEM demonstrated accurate representation of microbial relative abundance in both L-OLR and H-OLR when compared to the previous MFC study [1]. Furthermore, the sensitivity analysis of organic substrates, as illustrated in Fig. 4, provided further evidence that the model held true for both L-OLR and H-OLR conditions, spanning the acetate uptake rate range of 0.77 to 2.09 $\text{mmol }\text{g}_{\text{O}_{\text{2}}\text{-cell}}^{\text{-1}}\text{ }\text{h}^{\text{-1}}$ and the butyrate uptake rate range from 0.48 to 3.61 $\text{mmol }\text{g}_{\text{O}_{\text{2}}\text{-cell}}^{\text{-1}}\text{ }\text{h}^{\text{-1}}$. The analysis ensures the model's validity within the scope of the study.

**S2 Text Table 1 Condition-specific constraints for L-OLR and H-OLR condition.**

| **Reaction description** | **Reaction** | **L-OLR** | | **H-OLR** | |
| --- | --- | --- | --- | --- | --- |
|  |  | **Lower bound** | **Upper bound** | **Lower bound** | **Upper bound** |
| EX_but[u] | but[u] <=> | -2.4935 | -2.4935 | -1.5964 | -1.5964 |
| EX_ac[u] | ac[u] <=> | -1.6183 | -1.6183 | -1.2419 | -1.2419 |
| EX_sulfate[u] | so4[u] - | -0.0527 | -0.0527 | 0.078 | 0.078 |
| EX_sulfide[u] | HC00250[u] <=> | -0.0064 | -0.0064 | -0.1097 | -0.1097 |
| EX_h[u] | h[u] -> | -0.0004 | 0 | -0.007 | 0 |

The mmGEM also constrained by the constantly limiting exchange reactions based on 3 manners: (1) dual exchange – allowing both uptake and secretion, (2) secretion only, (3) confining the exchange flux to a specific value to mimic the MFC environment.

- Dual exchange:

Metabolic exchanges such as phosphate, ammonia, and photon, were allowed for both uptake and secretion. Although these compounds were not experimentally measured, they were essential for the model simulation. These metabolites were basically assumed to be abundant in the MFC environment.

- Secretion only:

Some metabolites were allowed only for secretion, assumed to be byproducts of the system. For instance, methane and H_2_ were not utilized by the microbial guilds in the mmGEM, necessitating their release into the outside environment to maintain mass balance.

- Confining to a specific value:

In case of constraining to a specific value, A metabolite, that is oxygen, was constrained followed the MFC system. As know that the MFC system is anoxic [3], the uptake of oxygen was constrained to a very low accordingly

All exchange constraints are listed in Table 2 in S2 Text.

**S2 Text Table 2 Exchange reactions constraints of mmGEM**

| **Reaction description** | **Reaction** | **Lower bound** | **Upper bound** |
| --- | --- | --- | --- |
| EX_1,2-Diacyl-sn-glycerol (dioctadecanoyl, n-C18:0)[u] | 12dgr180[u] -> | 0 | 1000 |
| EX_meso-2,6-Diaminoheptanedioate[u] | 26dap__M[u] -> | 0 | 1000 |
| EX_2-Demethylmenaquinone 8[u] | 2dmmq8[u] -> | 0 | 1000 |
| EX_2-Hydroxyphenylacetate[u] | 2hyoxplac[u] -> | 0 | 1000 |
| EX_2-Oxobutanoate[u] | 2obut[u] -> | 0 | 1000 |
| EX_L-dopa[u] | 34dhphe[u] -> | 0 | 1000 |
| EX_3-methyl-2-oxopentanoate[u] | 3mop[u] -> | 0 | 1000 |
| EX_4-Aminobenzoate[u] | 4abz[u] -> | 0 | 1000 |
| EX_4_Hydroxybenzoate_e0[u] | 4hbz[u] -> | 0 | 1000 |
| EX_(4-hydroxyphenyl)acetaldehyde[u] | 4hoxpacd[u] -> | 0 | 1000 |
| EX_4-hydroxyphenylacetate[u] | 4hphac[u] -> | 0 | 1000 |
| EX_5_Amino_6__5_phosphoribitylaminouracil[u] | 5aprbu[u] -> | 0 | 1000 |
| EX_Malonate[u] | HC00319[u] -> | 0 | 1000 |
| EX_apoprotein[u] | HC01161[u] <=> | -1000 | 1000 |
| EX_Thiosulfate[u] | HC01501[u] -> | 0 | 1000 |
| EX_Zinc[u] | HC02172[u] <=> | -1000 | 1000 |
| EX_L_alagly[u] | L_alagly[u] -> | 0 | 1000 |
| EX_Lcyst[u] | Lcyst[u] -> | 0 | 1000 |
| EX_Lkynr[u] | Lkynr[u] -> | 0 | 1000 |
| EX_Maltohexaose[u] | M02447[u] -> | 0 | 1000 |
| EX_acetoacetate[u] | acac[u] -> | 0 | 1000 |
| EX_N-acetyl-D-glucosamine[u] | acgam[u] -> | 0 | 1000 |
| EX_Acyl-CoA[u] | acoa[u] <=> | -1000 | 1000 |
| EX_Adenosine[u] | adn[u] -> | 0 | 1000 |
| EX_Adenosylcobalamin[u] | adocbl[u] <=> | -1000 | 1000 |
| EX_ala_L_Thr__L[u] | ala_L_Thr__L[u] -> | 0 | 1000 |
| EX_ala_L_asp__L[u] | ala_L_asp__L[u] -> | 0 | 1000 |
| EX_ala_L_gln__L[u] | ala_L_gln__L[u] -> | 0 | 1000 |
| EX_ala_L_glu__L[u] | ala_L_glu__L[u] -> | 0 | 1000 |
| EX_ala_L_leu__L[u] | ala_L_leu__L[u] -> | 0 | 1000 |
| EX_ala__D[u] | ala__D[u] -> | 0 | 1000 |
| EX_ala__L[u] | ala__L[u] -> | 0 | 1000 |
| EX_Allantoin[u] | alltn[u] -> | 0 | 1000 |
| EX_S_Adenosyl_L_methionine[u] | amet[u] -> | 0 | 1000 |
| EX_D_Arabinose[u] | arab__D[u] -> | 0 | 1000 |
| EX_arg__L[u] | arg__L[u] -> | 0 | 1000 |
| EX_asn__L[u] | asn__L[u] -> | 0 | 1000 |
| EX_asp__L[u] | asp__L[u] -> | 0 | 1000 |
| EX_(R)-3-hydroxybutyrate[u] | bhb[u] -> | 0 | 1000 |
| EX_butanesulfonate[u] | butso3[u] -> | 0 | 1000 |
| EX_calcium2+[u] | ca2[u] <=> | -1000 | 1000 |
| EX_Cob(I)alamin[u] | cbl1[u] -> | 0 | 1000 |
| EX_Cob(II)alamin[u] | cbl2[u] -> | 0 | 1000 |
| EX_Cadmium[u] | cd2[u] -> | 0 | 1000 |
| EX_cgly[u] | cgly[u] -> | 0 | 1000 |
| EX_Choline[u] | chol[u] -> | 0 | 1000 |
| EX_chorismate[u] | chor[u] -> | 0 | 1000 |
| EX_citrate[u] | cit[u] -> | 0 | 1000 |
| EX_chloride[u] | cl[u] <=> | -1000 | 1000 |
| EX_co2[u] | co2[u] -> | -1000 | 1000 |
| EX_coa[u] | coa[u] -> | 0 | 1000 |
| EX_cobalt2[u] | cobalt2[u] <=> | -1000 | 1000 |
| EX_Protoheme[u] | cpd00028[u] -> | 0 | 1000 |
| EX_D-Fructose[u] | cpd00082[u] -> | 0 | 1000 |
| EX_phenylacetate[u] | cpd00430[u] -> | 0 | 1000 |
| EX_5-hydroxy-L-tryptophan[u] | cpd00490[u] -> | 0 | 1000 |
| EX_D-glucarate(2-)[u] | cpd00609[u] -> | 0 | 1000 |
| EX_Methane[u] | cpd01024[u] -> | 0 | 1000 |
| EX_1_Aminopropan_2_ol[u] | cpd02039[u] <=> | -1000 | 1000 |
| EX_Vitamin_B12[u] | cpd03424[u] -> | 0 | 1000 |
| EX_Arbutin[u] | cpd03696[u] -> | 0 | 1000 |
| EX_Ferrichrome[u] | cpd03724[u] -> | 0 | 1000 |
| EX_Methanophenazine[u] | cpd08701[u] <=> | -1000 | 0 |
| EX_Dihydromethanophenazine[u] | cpd08702[u] <=> | 0 | 0 |
| EX_ACP[u] | cpd11493[u] <=> | -1000 | 1000 |
| EX_3-(n-Morpholino)Propanesulfonic Acid[u] | cpd11575[u] -> | 0 | 1000 |
| EX_L-alanyl-L-histidine[u] | cpd11584[u] -> | 0 | 1000 |
| EX_Glycyl-L-aspartate[u] | cpd11589[u] -> | 0 | 1000 |
| EX_Gly-Cys[u] | cpd15603[u] -> | 0 | 1000 |
| EX_Glycylleucine[u] | cpd15604[u] -> | 0 | 1000 |
| EX_Glycylphenylalanine[u] | cpd15605[u] -> | 0 | 1000 |
| EX_Glycyl-L-tyrosine[u] | cpd15606[u] -> | 0 | 1000 |
| EX_Corrinoid-Fe-S-proteins[u] | cpd26754[u] -> | 0 | 1000 |
| EX_cytosine[u] | csn[u] -> | 0 | 1000 |
| EX_cu2[u] | cu2[u] <=> | -1000 | 1000 |
| EX_Cyanate[u] | cynt[u] -> | 0 | 1000 |
| EX_cys__L[u] | cys__L[u] -> | 0 | 1000 |
| EX_Cytidine[u] | cytd[u] -> | 0 | 1000 |
| EX_2-deoxyadenosine[u] | dad_2[u] -> | 0 | 1000 |
| EX_5_Deoxyadenosine[u] | dad_5[u] -> | 0 | 1000 |
| EX_Iminoglycine[u] | dhgly[u] -> | 0 | 1000 |
| EX_dopaminium(1+)[u] | dopa[u] -> | 0 | 1000 |
| EX_dttp[u] | dttp[u] <=> | -1000 | 1000 |
| EX_ethanesulfonate[u] | eths[u] -> | 0 | 1000 |
| EX_fa[u] | fa[u] -> | 0 | 1000 |
| EX_fe2[u] | fe2[u] <=> | -1000 | 1000 |
| EX_fe3[u] | fe3[u] <=> | -1000 | 1000 |
| EX_FeIIIdicitrate[u] | fe3dcit[u] -> | 0 | 1000 |
| EX_Folate[u] | fol[u] <=> | -1000 | 1000 |
| EX_formate[u] | for[u] -> | 0 | 1000 |
| EX_fumarate[u] | fum[u] -> | 0 | 1000 |
| EX_gal__D[u] | gal__D[u] <=> | -1000 | 1000 |
| EX_D-Galactarate[u] | galct_D[u] -> | 0 | 1000 |
| EX_D-Galactonate[u] | galctn__D[u] -> | 0 | 1000 |
| EX_galactitol[u] | galt[u] -> | 0 | 1000 |
| EX_D-Glucosamine[u] | gam[u] -> | 0 | 1000 |
| EX_glycolaldehyde[u] | gcald[u] -> | 0 | 1000 |
| EX_glc__D[u] | glc__D[u] -> | 0 | 1000 |
| EX_gln__L[u] | gln__L[u] -> | 0 | 1000 |
| EX_glu__L[u] | glu__L[u] -> | 0 | 1000 |
| EX_gly[u] | gly[u] -> | 0 | 1000 |
| EX_gly_asn__L[u] | gly_asn__L[u] -> | 0 | 1000 |
| EX_gly_gln__L[u] | gly_gln__L[u] -> | 0 | 1000 |
| EX_gly_glu__L[u] | gly_glu__L[u] -> | 0 | 1000 |
| EX_gly_met__L[u] | gly_met__L[u] -> | 0 | 1000 |
| EX_gly_pro__L[u] | gly_pro__L[u] -> | 0 | 1000 |
| EX_glyb[u] | glyb[u] -> | 0 | 1000 |
| EX_glyc[u] | glyc[u] -> | 0 | 1000 |
| EX_glyclt[u] | glyclt[u] -> | 0 | 1000 |
| EX_gthox[u] | gthox[u] <=> | -1000 | 1000 |
| EX_gthrd[u] | gthrd[u] -> | 0 | 1000 |
| EX_gtp[u] | gtp[u] <=> | -1000 | 1000 |
| EX_h2co3[u] | h2co3[u] <=> | 0 | 1000 |
| EX_h2o2[u] | h2o2[u] -> | 0 | 1000 |
| EX_h2o[u] | h2o[u] -> | -1000 | 1000 |
| EX_Hexadecanoate (n-C16:0)[u] | hdca[u] -> | 0 | 1000 |
| EX_hexanesulfonate[u] | hexs[u] -> | 0 | 1000 |
| EX_his__L[u] | his__L[u] -> | 0 | 1000 |
| EX_histamium[u] | hista[u] -> | 0 | 1000 |
| EX_hv[u] | hv[u] <=> | -1000 | 1000 |
| EX_Tetrahydropteroyltri_L_glutamate[u] | hpglu[u] -> | 0 | 1000 |
| EX_Hypoxanthine[u] | hxan[u] -> | 0 | 1000 |
| EX_ile__L[u] | ile__L[u] -> | 0 | 1000 |
| EX_Inosine[u] | ins[u] -> | 0 | 1000 |
| EX_Isethionic acid[u] | isetac[u] -> | 0 | 1000 |
| EX_k[u] | k[u] <=> | -1000 | 1000 |
| EX_lac__D[u] | lac__D[u] -> | 0 | 1000 |
| EX_lac__L[u] | lac__L[u] -> | 0 | 1000 |
| EX_leu__L[u] | leu__L[u] -> | 0 | 1000 |
| EX_lys__L[u] | lys__L[u] -> | 0 | 1000 |
| EX_(S)-malate(2-)[u] | mal__L[u] -> | 0 | 1000 |
| EX_Maltose[u] | malt[u] -> | 0 | 1000 |
| EX_maltotriose[u] | malttr[u] -> | 0 | 1000 |
| EX_D-Mannose[u] | man[u] -> | 0 | 1000 |
| EX_D-mannose[u] | man__D[u] <=> | -1000 | 1000 |
| EX_met_L_ala__L[u] | met_L_ala__L[u] -> | 0 | 1000 |
| EX_met__D[u] | met__D[u] -> | 0 | 1000 |
| EX_met__L[u] | met__L[u] -> | 0 | 1000 |
| EX_L-Methionine Sulfoxide[u] | metox[u] -> | 0 | 1000 |
| EX_L-methionine-R-sulfoxide[u] | metox__R[u] -> | 0 | 1000 |
| EX_Methanofuran[u] | mfr_b[u] <=> | -1000 | 1000 |
| EX_mg2[u] | mg2[u] <=> | -1000 | 1000 |
| EX_5_Methyltetrahydropteroyltri_L_glutamate[u] | mhpglu[u] <=> | -1000 | 1000 |
| EX_mn2[u] | mn2[u] <=> | -1000 | 1000 |
| EX_D-Mannitol[u] | mnl[u] -> | 0 | 1000 |
| EX_mqn7[u] | mqn7[u] <=> | -1000 | 1000 |
| EX_mqn8[u] | mqn8[u] <=> | -1000 | 1000 |
| EX_methanesulfonate[u] | mso3[u] -> | 0 | 1000 |
| EX_na1[u] | na1[u] -> | -1000 | 1000 |
| EX_Nicotinate[u] | nac[u] -> | 0 | 1000 |
| EX_Nicotinamide[u] | ncam[u] <=> | -1000 | 1000 |
| EX_nh3[u] | nh3[u] <=> | -1000 | 1000 |
| EX_ni2[u] | ni2[u] <=> | -1000 | 1000 |
| EX_no2[u] | no2[u] -> | 0 | 1000 |
| EX_no3[u] | no3[u] -> | 0 | 1000 |
| EX_o2[u] | o2[u] <=> | -0.000095 | 1000 |
| EX_octadecanoate (n-C18:0)[u] | ocdca[u] -> | 0 | 1000 |
| EX_octadecenoate (n-C18:1)[u] | ocdcea[u] -> | 0 | 1000 |
| EX_Ornithine[u] | orn[u] -> | 0 | 1000 |
| EX_phe__L[u] | phe__L[u] -> | 0 | 1000 |
| EX_keto-phenylpyruvate[u] | phpyr[u] -> | 0 | 1000 |
| EX_pi[u] | pi[u] <=> | -1000 | 1000 |
| EX_PAN[u] | pnto__R[u] <=> | -1000 | 1000 |
| EX_propionate[u] | ppa[u] -> | 0 | 1000 |
| EX_ppi[u] | ppi[u] -> | 0 | 1000 |
| EX_pro__L[u] | pro_L[u] -> | 0 | 1000 |
| EX_Putrescine[u] | ptrc[u] -> | 0 | 1000 |
| EX_Pyridoxamine[u] | pydam[u] -> | 0 | 1000 |
| EX_Pyridoxal[u] | pydx[u] -> | 0 | 1000 |
| EX_Pyridoxine[u] | pydxn[u] <=> | -1000 | 1000 |
| EX_q8[u] | q8[u] <=> | -1000 | 1000 |
| EX_Riboflavin[u] | ribflv[u] -> | 0 | 1000 |
| EX_rmn__L[u] | rmn__L[u] <=> | -1000 | 1000 |
| EX_sulfur[u] | s[u] <=> | 0 | 1000 |
| EX_Salicin[u] | salcn[u] -> | 0 | 1000 |
| EX_D-glucitol[u] | sbt__D[u] -> | 0 | 1000 |
| EX_ser__D[u] | ser__D[u] -> | 0 | 1000 |
| EX_ser__L[u] | ser__L[u] -> | 0 | 1000 |
| EX_Siroheme[u] | sheme[u] -> | 0 | 1000 |
| EX_Spermidine[u] | spmd[u] <=> | -1000 | 1000 |
| EX_serotonin(1+)[u] | srtn[u] -> | 0 | 1000 |
| EX_succ[u] | succ[u] -> | 0 | 1000 |
| EX_sucr[u] | sucr[u] -> | 0 | 1000 |
| EX_sulfoacetate[u] | sula[u] -> | 0 | 1000 |
| EX_Taurine[u] | taur[u] -> | 0 | 1000 |
| EX_Thiamin[u] | thm[u] <=> | -1000 | 1000 |
| EX_Thiamine_phosphate[u] | thmmp[u] <=> | -1000 | 1000 |
| EX_thr__L[u] | thr__L[u] -> | 0 | 1000 |
| EX_Thymidine[u] | thymd[u] -> | 0 | 1000 |
| EX_Trehalose[u] | tre[u] -> | 0 | 1000 |
| EX_trp__L[u] | trp__L[u] -> | 0 | 1000 |
| EX_tryptaminium[u] | trypta[u] -> | 0 | 1000 |
| EX_tetradecanoate (n-C14:0)[u] | ttdca[u] -> | 0 | 1000 |
| EX_tyraminium[u] | tym[u] -> | 0 | 1000 |
| EX_tyr__L[u] | tyr__L[u] -> | 0 | 1000 |
| EX_Guanine[u] | gua[u] -> | 0 | 0 |
| EX_uracil[u] | ura[u] -> | 0 | 1000 |
| EX_Uridine[u] | uri[u] -> | 0 | 1000 |
| EX_val__L[u] | val__L[u] -> | 0 | 1000 |
| EX_Xanthine[u] | xan[u] -> | 0 | 1000 |
| EX_h2[u] | h2[u] -> | 0 | 1000 |

**Parameterization of mmGEM**

The mmGEM parameterization was done at the microbial interaction space to optimize the relationship of SRB, MET, and SOB microbial guilds. The process was performed for 2 objectives: (1) to mimic the simulated microbial relative abundances of SRB, MET, and SOB in the L-OLR experiment, and (2) to replicate their growth response to increased OLR as observed in the MFC system study. The parameterization process was done after the refinement of mmGEM metabolic network, which ensured the accurate incorporation and refinement of key metabolic functions such as sulfate reduction, methanogenesis, and sulfide oxidation. Our findings highlighted that metabolic cross-feeding among microbial guilds significantly influenced community structure and responses. Specifically, we parameterized the exchange capacities of three metabolites—H^+^, acetate, and sulfide—across six exchange reactions (S2 Text Table 3). These cross-feeding metabolic capacities were applied to constrain mmGEM, while the rest of the exchange reactions between microbial compartments were freely exchanges. The details of individual parameterization step are shown below.

**Table 3 Parameterization of the microbial exchange capacity in mmGEM.**

| **Exchange fluxes** | **Exchange reactions** | **Types of constraints** | **Ranges of constraint^*^** |
| --- | --- | --- | --- |
| H^+^ exchange of SRB ($v_{H^{+}}^{SRB}$) | SRB_h[e] <=> h_ex[u] | UB | 1$\times$10^-1^ – 1$\times$10^-5^ |
| H^+^ exchange of MET ($v_{H^{+}}^{MET}$) | MET_h[e] <=> h_ex[u] | UB | 1$\times$10^-1^ – 1$\times$10^-5^ |
| Acetate exchange of SOB ($v_{Acetate}^{SOB}$) | SOB_ac[e] <=> ac[u] | LB | -1.50 – -1.75 |
| Acetate exchange of MET ($v_{Acetate}^{MET}$) | MET_ac[e] <=> ac[u] | UB | -10.50 – -11.55 |
| Sulfide exchange of SRB ($v_{Sulfide}^{SRB}$) | SRB_H2S[e] <=> H2S[u] | UB | 5.87 – 6.28 |

Note:

* Range of constraint flux that model still predicts similar microbial profile in the unit ($\text{mmol} \text{g}_{\text{O}_{\text{2}}\text{-cell}}^{\text{-1}}\text{ }\text{h}^{\text{-1}}$).

LB and UB are lower bound and upper bound, respectively.

For optimizing microbial growth, the H^+^ exchange capacity ($v_{{ex(H}^{+})}^{k}$) of SRB and MET were parameterized by observing simulated microbial relative abundances and comparing them to the experiment conducted by Sriwichai et al. (2024) [1]. As shown in S2 Fig, we found that leaving ($v_{{ex(H}^{+})}^{k}$) unconstrained caused excessive growth of SOB, as SRB and MET consistently released high fluxes of H^+^ to SOB. This additional H^+^ supported ATP production in SOB, leading the community structure to deviate from the experimental results. Moreover, it is known that environmental H^+^ concentration (pH) is a crucial parameter for microbial metabolic function. For instance, low pH conditions reduce methanogenesis in MET [4], while some microbial species can uptake H^+^ to support their ATP production [5]. Therefore, this variable was parameterized by performing an analysis on the H^+^ secretion capacity of SRB and MET ($\text{UB}$ of $v_{{ex(H}^{+})}^{SRB}$ and $v_{{ex(H}^{+})}^{MET}$), while observing simulated microbial relative abundances and calculating the average percent error compared to the experiment. The result indicated that within the range of the upper bounds of $\text{UB}$ of $v_{{ex(H}^{+})}^{SRB}$ and $v_{{ex(H}^{+})}^{MET}$ of 1$\times$10^-1^ – 1$\times$10^-5^ $\text{mmol} \text{g}_{\text{O}_{\text{2}}\text{-cell}}^{\text{-1}}\text{ }\text{h}^{\text{-1}}$, the percent error of microbial relative abundances decreased (S2 Fig). This analysis suggested that the MFC microbiome is unlikely to share H^+^ among participants. Consequently, the upper bound of $v_{{ex(H}^{+})}^{SRB}$ and $v_{{ex(H}^{+})}^{MET}$ of mmGEM was constrained at 1$\times$10^-5^ $\text{mmol} \text{g}_{\text{O}_{\text{2}}\text{-cell}}^{\text{-1}}\text{ }\text{h}^{\text{-1}}$.

Subsequently, the mmGEM was further parameterized to optimize microbial growth response to substrates in varying OLR environment that two main steps were performed: (1) performing a sensitivity analysis on organic metabolite uptakes (butyrate and acetate) in the MFC system to evaluate their effects on the growth of microbial guilds and (2) further parameterizing the major metabolic cross-feedings influenced by the increase in these organic metabolites. Sensitivity analysis of these interactions was conducted, while the trajectories of microbial growth rate under varying OLR condition were observed.

Previous MFC studies suggested that during increasing OLR, the SOB proportion decreased while the MET relative abundance drastically increased (S1 Table) [1]. The influx rate of organic compounds affected microbial composition; however, it is still unclear which specific organic metabolites caused this phenomenon in the MFC system. Therefore, we first evaluated the effects of butyrate and acetate uptake rates on microbial growth. The mmGEM simulated varying community uptake rates of butyrate and acetate. From S2 Text Fig 1A, the analysis showed that butyrate had a more significant influence on microbial growth rates than acetate (Fig 1B in S2 Text). Specifically, the growth rate of SOB increased considerably with higher butyrate uptake, with a much steeper slope compared to MET. It was evident that butyrate was a more critical contributor to microbial growth. However, the mmGEM still did not accurately respond to butyrate uptake. It was hypothesized that this inaccuracy was due to incorrect microbial interactions related to the community butyrate uptake.


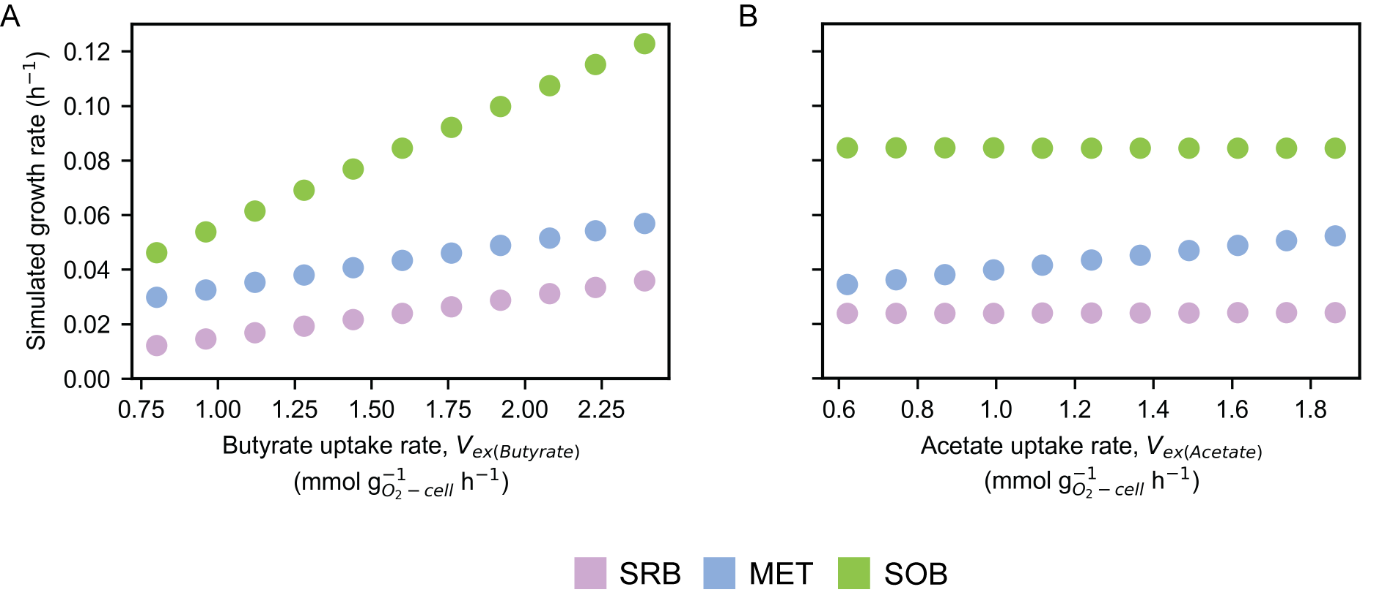


**Fig 1 Simulated microbial growth rates during increasing community butyrate and acetate uptake rates.** Sensitivity analysis of (A) butyrate and (B) acetate uptake rate was conducted to observe microbial growth rate response.

Within the scope of mmGEM, SRB is the sole microbial guild metabolizing butyrate. SRB typically metabolizes butyrate with sulfate, producing acetate and sulfide as products ($\mathrm{Butyrate}^{-}+0.5 \mathrm{SO}_{4}^{2-}\to2 \mathrm{Acetate}^{-}+{0.5 HS}^{-}+{0.5 H}^{+}$) [6]. This aligns with the simulation results showing SRB producing acetate and sulfide at high flux values. In addition, by considering the ecological role of these compounds in wastewater treatment, acetate and sulfide serve as metabolic currencies that transfer energy to other microbial participants in the community [7]. Sharing these metabolites, particularly in the multi-step degradation of compounds (syntrophic interaction), is common in wastewater treatment [8]. Therefore, these metabolic interactions were further investigated their effect to the microbial growth responses under increasing OLR condition.

In the current simulation, MET’s growth is less competitive than SOB (S2 Text Fig 1A). Thus, the minimum acetate uptake capacity of MET ($\mathrm{LB}$ of $v_{ex(Acetate)}^{MET}$) and the maximum acetate uptake capacity of SOB ($\mathrm{UB}$ of $v_{ex(Acetate)}^{SOB}$) were specifically focused in this step. Constraining these exchange capacities was intended to mimic acetate competition between MET and SOB in the studied MFC condition. The sensitivity analysis of these exchange capacities was performed during increasing community butyrate uptake rates. The original metabolite exchange capacities were derived from relationship between their fluxes and microbial relative abundances in the L-OLR condition (refer to Equation 1 in Materials and Methods; $v_{ex(Acetate)}^{k}=\frac{V_{ex(Acetate)}^{k}}{X^{k}}$). The trajectory of the MET growth rate, represented by slope, was observed during the analysis. A high slope of MET growth was expected based on previous MFC work observations. S2 Text Fig 2A shows that constraining the $\mathrm{LB}$ of $v_{ex(Acetate)}^{MET}$ at -10.50 – -11.55 $\text{mmol} \text{g}_{\text{O}_{\text{2}}\text{-cell}}^{\text{-1}}\text{ }\text{h}^{\text{-1}}$and $\mathrm{UB}$ of $v_{ex(Acetate)}^{SOB}$ at -1.5 – -1.75 $\text{mmol} \text{g}_{\text{O}_{\text{2}}\text{-cell}}^{\text{-1}}\text{ }\text{h}^{\text{-1}}$ gave the highest slope. Subsequently, the mmGEM was constrained with the $\mathrm{LB}$ of $v_{ex(Acetate)}^{MET}$ and $\mathrm{UB}$ of $v_{ex(Acetate)}^{SOB}$ at -10.5 and -1.5 $\text{mmol} \text{g}_{\text{O}_{\text{2}}\text{-cell}}^{\text{-1}}\text{ }\text{h}^{\text{-1}}$, respectively. The results showed better microbial growth rate response under dynamic community butyrate uptake rates (Fig 2B in S2 Text). The increase in SOB’s growth rate was slower than that of MET, which allowed MET to dominate the community at later stages of the increasing butyrate uptake.


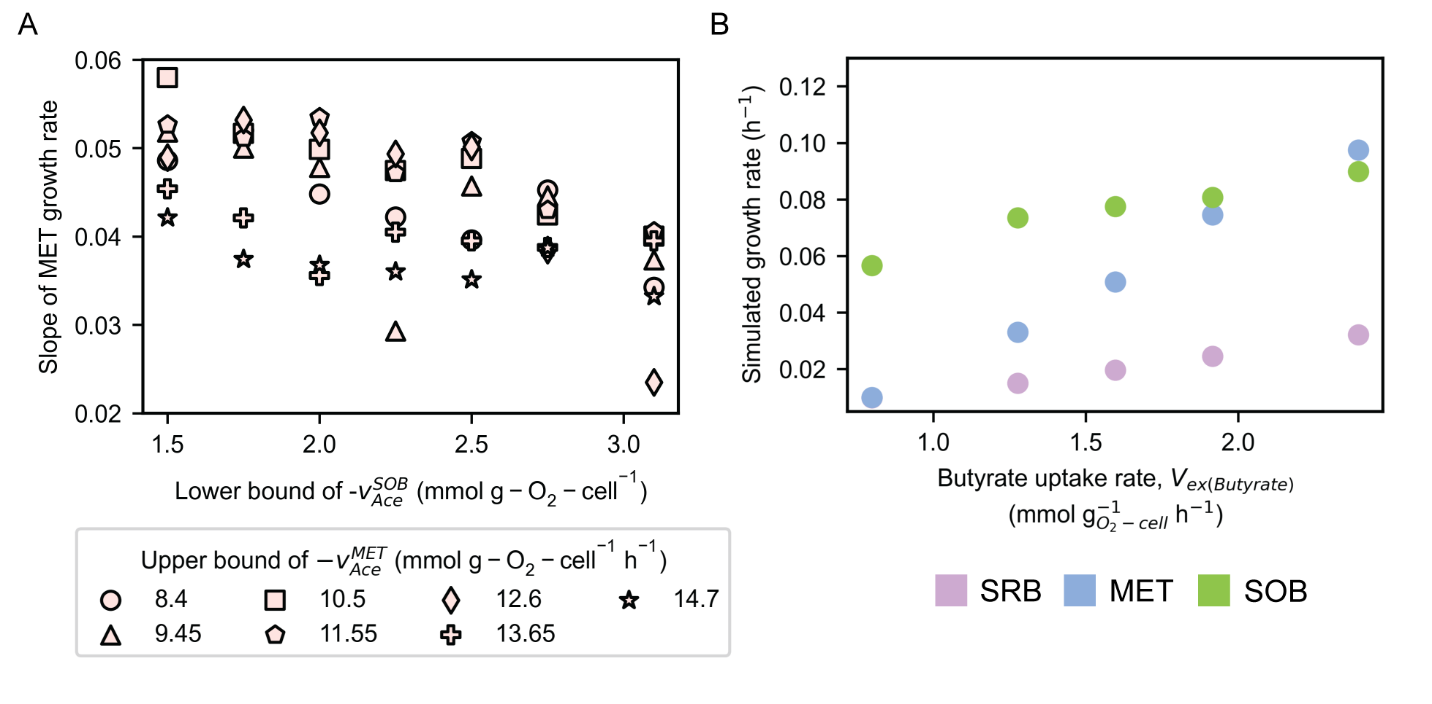


**Fig 2 Parameterization of the minimum of acetate uptake capacity of MET (UB of** $\boldsymbol{v}_{\boldsymbol{Acetate}}^{\boldsymbol{MET}}$**) and maximum acetate uptake of SOB (LB of** $\boldsymbol{v}_{\boldsymbol{Acetate}}^{\boldsymbol{SOB}}$**).** (A) The slope of the MET growth rate during the butyrate uptake rate was increased in different acetate capacities of MET and SOB. (B) The dynamic growth rate of microbial guilds during increased butyrate uptake rate when LB of $v_{Acetate}^{SOB}$ and UB of $v_{Acetate}^{MET}$ were constrained at -1.5 and -10.5 $\text{mmol} \text{g}_{\text{O}_{\text{2}}\text{-cell}}^{\text{-1}}\text{ }\text{h}^{\text{-1}}$ respectively.

Subsequently, the effect of sulfide secretion from SRB was further investigated, focusing on the variable of maximum sulfide secretion capacity of SRB (UB of $v_{Sulfide}^{SRB}$). S2 Text Fig 3A showed that while the sulfide secretion capacity of SRB could affect the slope of MET’s growth, it had a relatively small impact compared to the acetate exchange capacities. As expected, this parameter more significantly influenced SOB growth rates. It was found that constraining the UB of $v_{Sulfide}^{SRB}$ prevented a high growth rate of SOB, particularly within the range of 5.87 – 6.28 $\text{mmol} \text{g}_{\text{O}_{\text{2}}\text{-cell}}^{\text{-1}}\text{ }\text{h}^{\text{-1}}$. With the mmGEM constrained at UB of $v_{Sulfide}^{SRB}$ = 6.28 $\text{mmol} \text{g}_{\text{O}_{\text{2}}\text{-cell}}^{\text{-1}}\text{ }\text{h}^{\text{-1}}$, the SOB’s growth rate was even lower when increasing OLR (S2 Text Fig 3B).

**
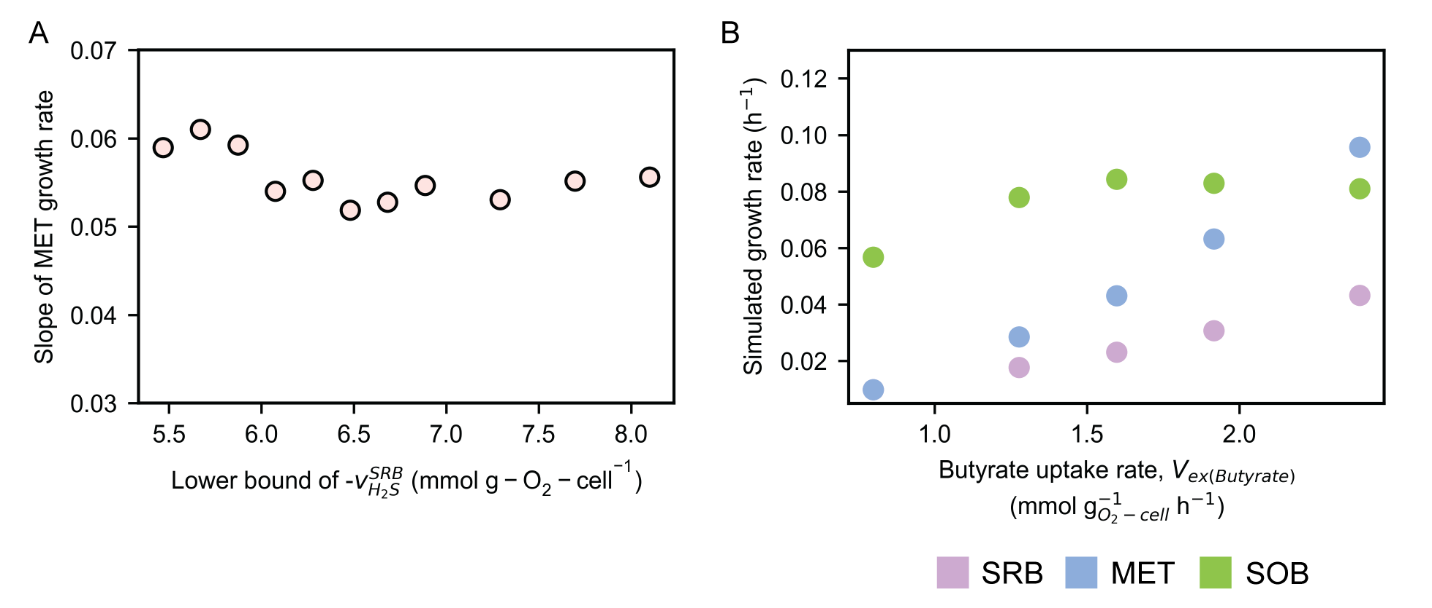
**

**Fig 3 Parameterization of the maximum sulfide secretion capacity of SRB (UB of** $\boldsymbol{v}_{\boldsymbol{Sulfide}}^{\boldsymbol{SRB}}$**).** (A) The slope of the MET growth rate during the butyrate uptake rate was increased in different sulfide capacities of SRB. (B) The dynamic growth rate of microbial guilds during increased butyrate uptake rate when UB of $v_{Sulfide}^{SRB}$were constrained at 6.28 $\text{mmol} \text{g}_{\text{O}_{\text{2}}\text{-cell}}^{\text{-1}}\text{ }\text{h}^{\text{-1}}$.

**Reference**

1. Sriwichai N, Sangcharoen R, Saithong T, Simpson D, Goryanin I, Boonapatcharoen N, et al. Optimization of microbial fuel cell performance application to high sulfide industrial wastewater treatment by modulating microbial function. PLoS One. 2024;19. doi:https://doi.org/10.1371/journal.pone.0305673

2. Lee HS, Parameswaran P, Kato-Marcus A, Torres CI, Rittmann BE. Evaluation of energy-conversion efficiencies in microbial fuel cells (MFCs) utilizing fermentable and non-fermentable substrates. Water Res. 2008;42: 1501–1510. doi:10.1016/j.watres.2007.10.036

3. Logan BE, Rabaey K. Conversion of wastes into bioelectricity and chemicals by using microbial electrochemical technologies. Science. 2012;337: 686–690. doi:10.1126/science.1217412

4. Sun M, Liu B, Yanagawa K, Ha NT, Goel R, Terashima M, et al. Effects of low pH conditions on decay of methanogenic biomass. Water Res. 2020;179. doi:10.1016/j.watres.2020.115883

5. Albert LS, Brown DG. Variation in bacterial ATP concentration during rapid changes in extracellular pH and implications for the activity of attached bacteria. Colloids Surf B Biointerfaces. 2015;132: 111–116. doi:10.1016/j.colsurfb.2015.05.020

6. Muyzer G, Stams AJM. The ecology and biotechnology of sulphate-reducing bacteria. Nat Rev Microbiol. 2008;6: 441–454. doi:10.1038/nrmicro1892

7. Morris BEL, Henneberger R, Huber H, Moissl-Eichinger C. Microbial syntrophy: Interaction for the common good. FEMS Microbiol Rev. 2013;37: 384–406. doi:10.1111/1574-6976.12019

8. Krohn C, Khudur L, Dias DA, van den Akker B, Rees CA, Crosbie ND, et al. The role of microbial ecology in improving the performance of anaerobic digestion of sewage sludge. Front Microbiol. 2022;13. doi:10.3389/fmicb.2022.1079136
