# Supplementary figures and images for: Metabolic cross-feeding interactions modulate the dynamic community structure in microbial fuel cell under variable organic loading wastewaters
Source: PLoS Comput Biol. 2024 Oct 17;20(10):e1012533. doi: 10.1371/journal.pcbi.1012533 (PMC11521316; doi:10.1371/journal.pcbi.1012533)

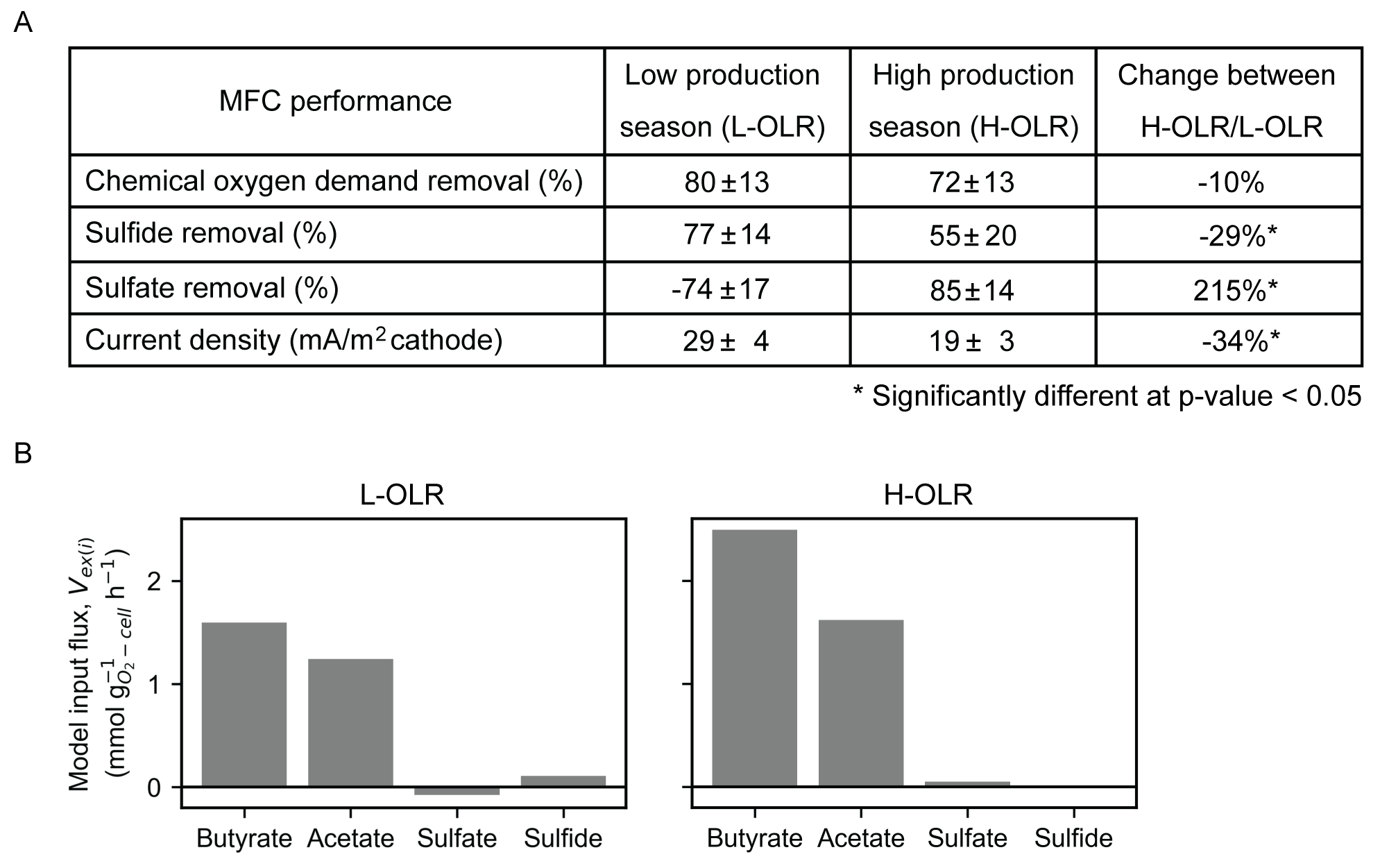

Supplement: S1 Fig — A, Performance of MFC from previous experiments under low organic loading (L-OLR) and high organic loading (H-OLR) conditions [5]. B, The model input flux (Vex(i)) for representing the MFC specific conditions in L-OLR and H-OLR. Vex(i) is an exchange reaction flux of the metabolite i in community compartment (COM) Positive (+) and negative (-) exchange fluxes indicate metabolite influx (+) and efflux (-) for COM. (TIF) [file pcbi.1012533.s007.tif]

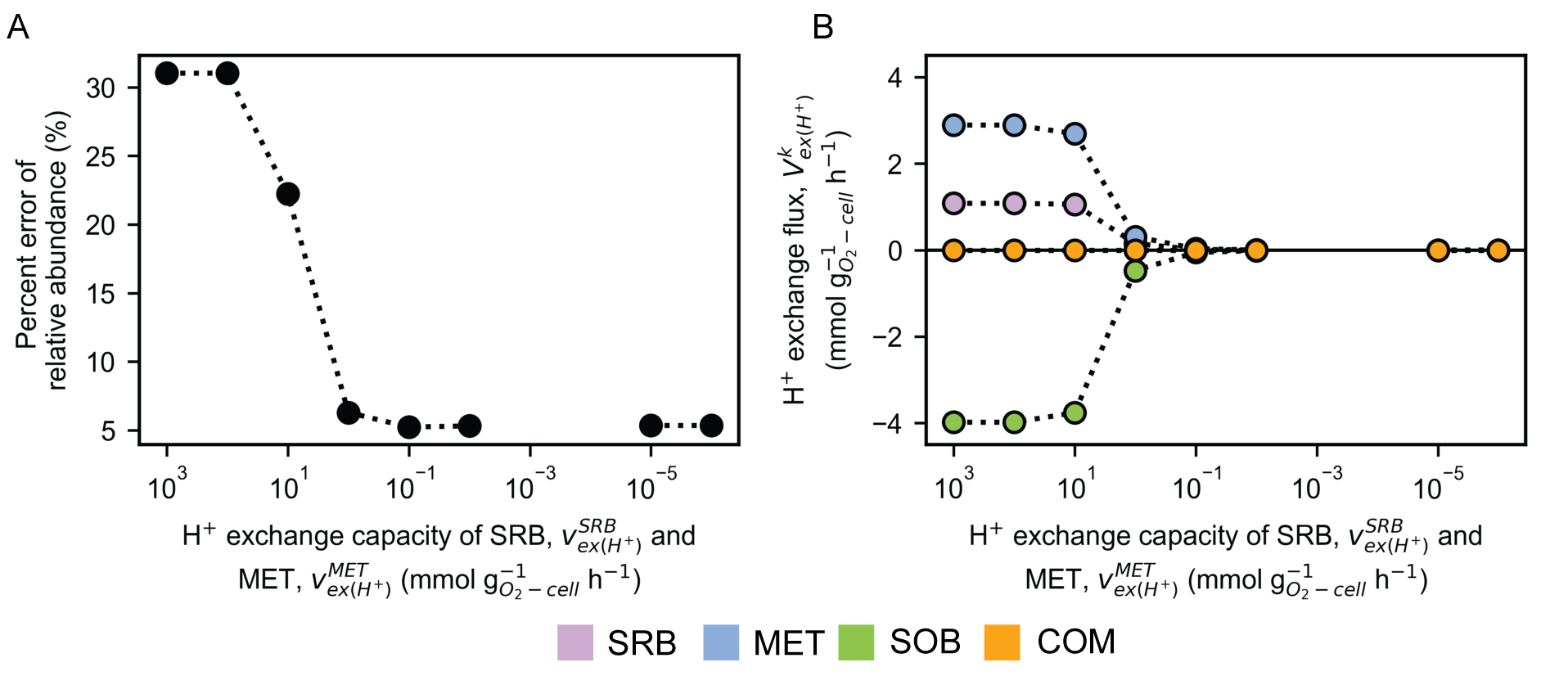

Supplement: S2 Fig — A, Average percent error of microbial relative abundances, including sulfate-reducing bacteria (SRB), methanogens (MET), and sulfide-oxidizing bacteria (SOB), between model simulation and experimental data in the low organic loading condition (L-OLR) and B, H+ exchange flux (Vex(H+)k) while perturbing H+ exchange capacities of SRB (vex(H+)SRB) and MET (vex(H+)MET).Vex(H+)k is an exchange reaction flux of H+ in community compartment (COM) and the microbial group k including SRB, MET, and SOB. Positive (+) and negative (-) exchange fluxes indicate metabolite secretion (+) and consumption (-) for microbial compartments, while representing metabolite influx (+) and efflux (-) for COM. (TIF) [file pcbi.1012533.s008.tif]

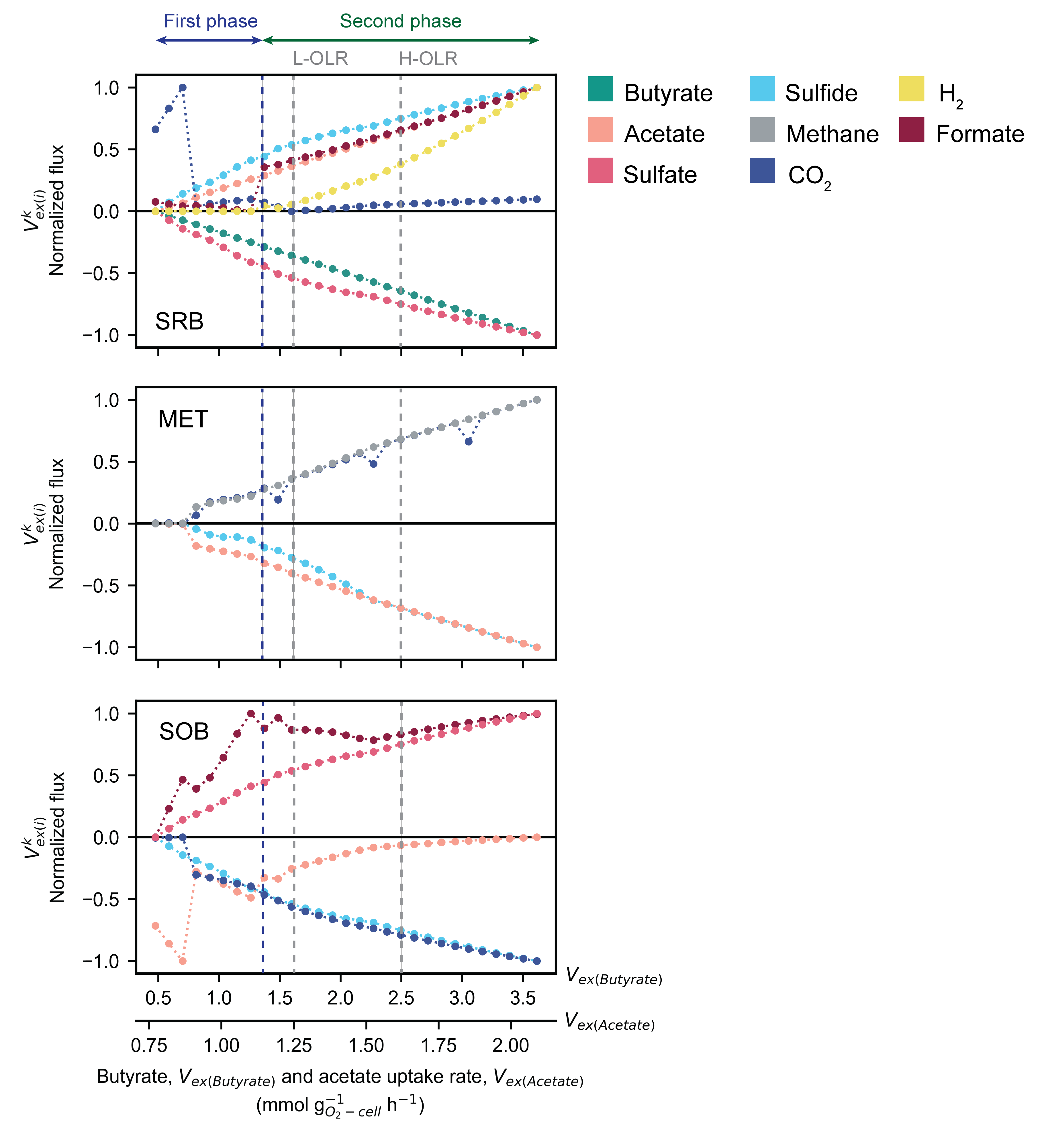

Supplement: S3 Fig — Normalized exchange fluxes (Vex(i)k) of sulfate-reducing bacteria (SRB), methanogens (MET), and sulfide-oxidizing bacteria (SOB) under increasing organic concentration. The fluxes of an exchange reaction across the increasing organic concentration were normalized by min-max normalization. The first and second phases are separated by a dashed-blue line. The first phase is defined between 0.48 and 1.26 mmol gO2‐cell‐1h‐1 for butyrate uptake rate and 0.77 to 1.10 mmol gO2‐cell‐1h‐1 for acetate uptake rate. The second phase ranges from 1.37 to 3.61 and 1.15 to 2.09 mmol gO2‐cell‐1h‐1 for butyrate and acetate uptake rates, respectively. Dashed-grey lines represent points of the organic concentration, equal to low organic loading condition (L-OLR) and high organic loading conditions (H-OLR). vex(i)k is exchange capacity flux of the metabolite i of microbial group k including SRB, MET, and SOB. Positive (+) and negative (-) normalized exchange capacity fluxes indicate metabolite secretion (+) and consumption (-) for microbial compartments. (TIF) [file pcbi.1012533.s009.tif]

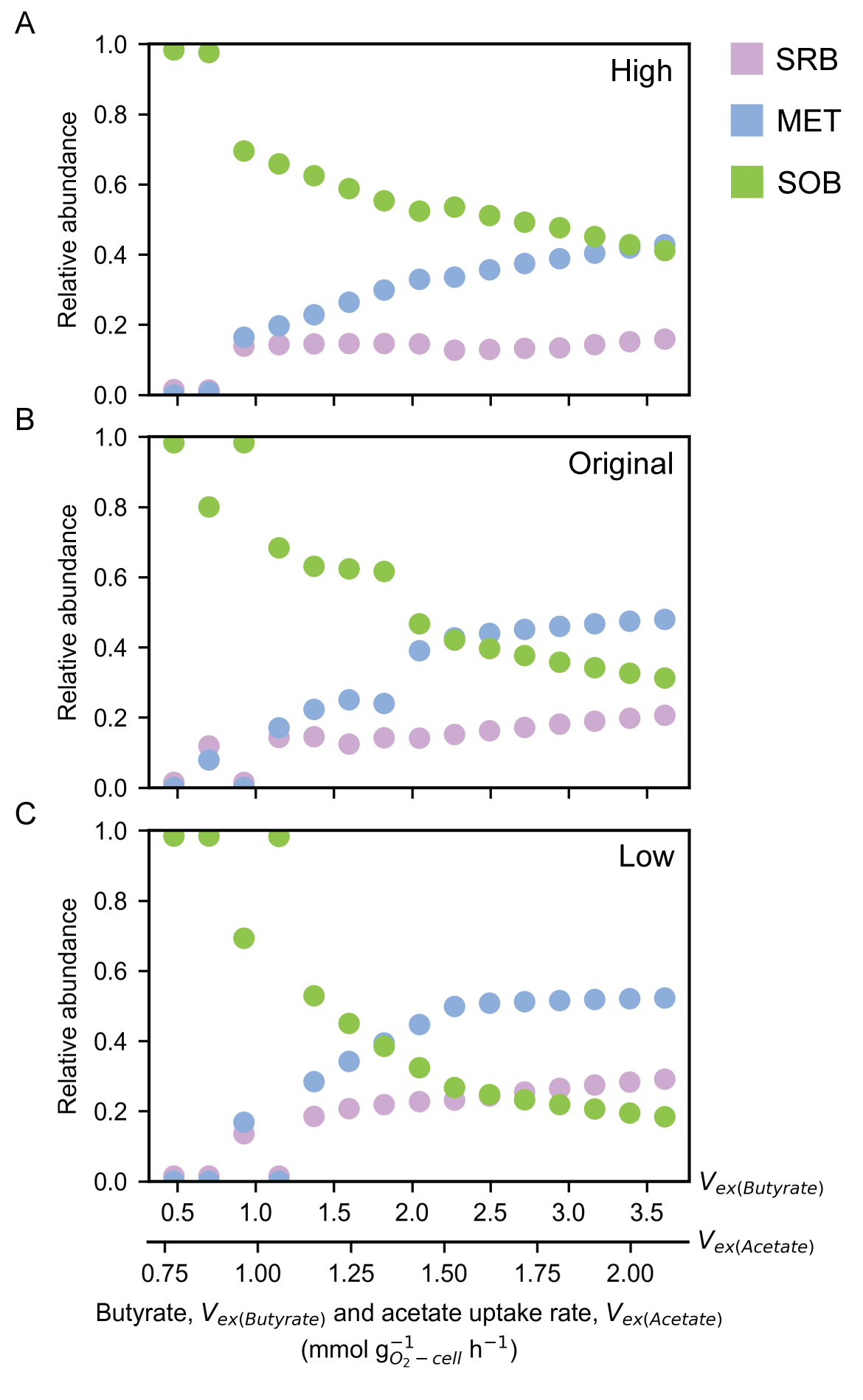

Supplement: S4 Fig — The growth patterns of sulfate-reducing bacteria (SRB), methanogens (MET), and sulfide-oxidizing bacteria (SOB), are from (A) high, (B) original, and (C) low exchange capacities of S cycling (vex(Sulfide)SRB) and acetate cross-feeding (vex(Acetate)SOB) between SRB and SOB. (TIF) [file pcbi.1012533.s010.tif]
